# Iron Single-Atom Catalysts Anchored on Defect-Engineered N‑Doped Graphene Reveal an Interplay between CO_2_ Reduction Activity and Stability

**DOI:** 10.1021/acssuschemeng.5c01417

**Published:** 2025-05-28

**Authors:** Dagmar Zaoralová, Rostislav Langer, Michal Otyepka

**Affiliations:** † IT4Innovations, VSB—Technical University of Ostrava, 17. Listopadu 2172/15, 708 00 Ostrava-Poruba, Czech Republic; ‡ Regional Centre of Advanced Technologies and Materials, The Czech Advanced Technology and Research Institute (CATRIN), 48207 Palacký University Olomouc, Šlechtitelů 27, 779 00 Olomouc, Czech Republic

**Keywords:** Single-atom catalysis, Stability, Activity, SAC, CO_2_RR

## Abstract

The precise engineering of vacancies in nitrogen-doped graphene (NG) presents a promising strategy for stabilizing metal single-atom catalysts (SACs) and tuning their catalytic performance. We explore the role of vacancies in NG for stabilizing iron-based SACs (Fe-SACs) by using density functional theory (DFT). First, we examine the stability of various vacancy types in graphene and NG supports, addressing the question of preferential formation of specific structural defects as potential sites for metal binding. We reveal simple rules governing the stability of vacancies and show that nitrogen doping can bring about vacancy healing. We identify preferred binding sites for Fe atoms/ions, specifically single and double vacancies, and analyze how the nitrogen-doping pattern in a vacancy affects the interaction of Fe with the SAC support. The results show that the positions of nitrogen(s) and the local charge environment significantly influence the stability of the Fe-SACs. Notably, some Fe@NG configurations, although not the most thermodynamically stable, exhibit enhanced catalytic performance, particularly for a CO_2_ reduction reaction (CO_2_RR). These findings offer valuable insights into vacancy engineering as a strategy for designing high-performance Fe-SACs and emphasize the interplay among vacancy types, nitrogen concentration, and catalyst stability in driving the catalytic behavior.

## Graphical abstract

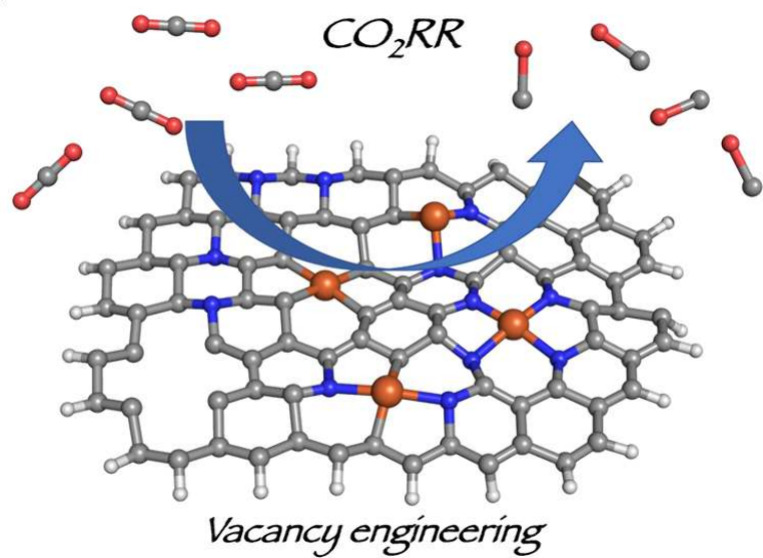

## Introduction

In the field of catalysis, the emergence of single-atom catalysts (SACs) marks a substantial advancement, introducing enhanced efficiency and precision in chemical reactions.^1^ SACs, by virtue of their isolated active sites, maximize atomic utilization and offer distinct advantages over traditional heterogeneous and homogeneous catalysts, including enhanced catalytic activity, improved selectivity, and ease of catalyst separation from the reaction mixture.^2^ These properties make SACs particularly valuable in applications where control at the molecular level can lead to more sustainable and economically viable processes.^3^ Despite the promise of SACs, their synthesis raises substantial challenges, primarily due to the intricacies of controlling atomic dispersion and maintaining stability under reaction conditions.^4–6^ The synthesis typically involves the reaction of transition metal (TM) salts with carefully engineered supports, such as defective and doped materials, including graphene altered at the atomic level to introduce specific functional properties.^7–11^ These supports play a crucial role in the stabilization of the active TM atoms, thereby significantly modulating the SAC performance.^12–14^

Nitrogen-doped graphene (NG) stands out as a highly valuable support in catalysis, thanks to its ability to modify electronic properties and create active sites for SACs.^15–17^ Vacancies and defects in NG serve as anchoring points for TMs, forming SACs active sites that define catalytic activity. However, several challenges persist, such as achieving precise control over vacancy formation and determining which types of vacancies are most effective under real catalytic conditions. Additionally, understanding the precise control of the metal environment, along with the influence of charge states, spin configurations, and reaction conditions, remains critical for optimizing the design of these systems. Iron stands out among TM elements due to its high abundance in the Earth’s lithosphere, ease of processing, and sustainability.^18^ Iron incorporated into the NG lattice (Fe@NG) leads to a wide class of SACs, which can be utilized in many important chemical reactions and processes involved in sustainable applications such as oxygen reduction reaction (ORR),^19^ and carbon dioxide reduction reaction (CO_2_RR).^20^ CO_2_RR is an important process involved in the transformation of carbon dioxide to valuable chemicals and fuels.^21–24^

However, the targeted rational design of SACs for specific reactions, including CO_2_RR, remains an important challenge in this field mostly due to the lack of understanding of the structure–activity relationships that govern their function.^18,25,26^ Theoretical calculations have proved to be indispensable tools in this context, offering unique insights into the electronic, geometric, and energetic aspects of SACs.^27–30^ The computational studies not only rationalize the underlying mechanisms of catalyst operation but also predict the influence of variables, such as the oxidation state of the metal, the nature of the dopants in the support, and the environmental conditions of the reaction. Through these insights, theoretical modeling aids in circumventing the empirical trial-and-error approach, steering the development of SACs toward systems with optimized reactivity and stability.^21,31^ However, despite progress in this field, many fundamental questions concerning the understanding of properties and operation of SACs, e.g., focused on the trade-off between catalyst stability and activity, remain unanswered.

In this study, we address this challenge by engineering Fe@NG supports to enhance both aspects simultaneously, i.e., the balance between stability and catalytic activity. First, we investigated the stability of defects in graphene and NG to systematically elucidate preferred binding sites for SACs. We explored the formation energies of various vacancies in graphene and NG, identifying the preferred vacancy types and the factors that influence their formation. We extend our investigation to examine how these materials interact with iron across its major oxidation states (0, II, and III) and how these interactions are influenced by the spin state and the solvent. Finally, we investigated energy profiles along CO_2_RR to get insights into the relationships between catalysts stability and activity. Our results show that the energy profile of this catalytic process is significantly modulated by the nature of the Fe@NG active site and identify the most catalytically efficient configurations. Thus, through controlled defect engineering, we tune the electronic environment of Fe active sites, modulating durability and reactivity. These findings offer valuable insights for optimizing the SAC performance and advancing their application in sustainable energy technologies.

## Computational Details

Two types of models of graphene and NG supports were considered, namely, finite models derived from computationally accessible polycyclic aromatic hydrocarbons and infinite 2D models attained by applying periodic boundary conditions (PBC).^32–34^ This dual approach provides a comprehensive and accurate description of local and lattice/bulk effects. Finite models are crucial for accurately capturing charged ions, multiplicity effects, charge distribution, and local interactions, which are key to understand the impact of functionalization at specific sites. On the other hand, the PBC calculations allow for the simulation of an infinite (N-doped) graphene sheet, effectively eliminating edge effects and providing insights into the bulk as well as intrinsic properties of the material.

PBC calculations were performed by using spin-polarized density functional theory (SP-DFT) with the Perdew, Burke, and Ernzerhof (PBE) functional^35^ and projected augmented wave potentials (PAW) representing atomic cores as implemented in the Vienna ab initio simulation package (VASP).^33,36–38^ For the calculations of nitrogen and/or iron atom incorporation into vacancies (Figure S14), Grimme’s D3 correction^39^ was applied. Test calculations (Table S1) for the system containing the DV­(5-8-5)-4N defect (Figure S12a) confirmed that a plane-wave basis set with a 400 eV cutoff and Brillouin zone integration using a 3 × 3 × 1 Γ-centered Monkhorst–Pack k-point mesh per conventional 6 × 6 triclinic cell (72 carbon atoms) provide sufficient accuracy. Therefore, this setup was used for further calculations. In the case of larger vacancies such as DV­(555-777) and DV­(555-6-777) (Figure S1b,c) an 8 × 8 triclinic cell containing 126 carbon atoms was used. A slab model with ca. 30 Å of vacuum width between layers of graphene was used. Geometric optimization of atom positions as well as lattice parameters was performed while keeping the volume of the supercell constant. The defect labeling used in this study is based on the work of Banhart et al.^40^ A clearer explanation is provided in Figure S2.

The finite models of Fe-doped defective graphene derived from coronene C_24_H_12_ (Figure S3a,b) with various nitrogen-doping patterns were optimized by using three different setups as implemented in Gaussian:^41^ (i) the long-range corrected ωB97X-D functional^42^ including an empirical van der Waals correction coupled with the def2-SVP basis set^43^ (method 1). (ii) The hybrid B3LYP functional^44–46^ with the Grimme empirical correction D3.^47^ The main group elements (C, N, H) were described with the def2-SVP basis set, while the Fe atoms/ions were treated with LANL2DZ basis set and respective effective core potential^48,49^ (method 2). (iii) The unrestricted version of the long-range-corrected ωB97X-D functional with empirical van der Waals correction coupled with the correlation-consistent polarized double-ζ valence basis set, cc-pVDZ^50^ (method 3), which became the main computational setup used in this study. Subsequently, a larger model derived from circumcoronene C_54_H_18_ (Figure S3c,d) was also studied by method 3. To account for the solid-state synthesis and the effect of solvents, the calculations were performed for the gas phase and water solvent, which was simulated using the universal continuum solvation model based on electron density (SMD).^51^

In the PBC calculations, the thermodynamic stability of individual structures of (nitrogen-doped) graphene containing defects or vacancies was expressed as the formation energy (*E*_form_) calculated as

1 $$E_{\mathrm{form}}=E_{\mathrm{defect}}+nE_{C}-E_{\mathrm{graphene}}-\frac{m}{2}E_{N_{2}}$$

where *E*_defect_ is the total energy of the (nitrogen-doped) graphene supercell containing defect or vacancy, *n* is the number of carbon atoms that were removed from the unperturbed graphene lattice to form a vacancy, *E*_C_ is the total energy of carbon atom in unperturbed graphene, *E*_graphene_ is the total energy of the unperturbed graphene supercell, *m* is the number of nitrogen atoms incorporated in the graphene lattice, and *E*_N_2__ is the total energy of N_2_ molecule.

In the finite model calculations, the interaction energies of Fe atoms/ions in Fe@NG, *E*_int_, were evaluated as

2 $$E_{\mathrm{int}}=E_{\mathrm{NG}+\mathrm{Fe}}-E_{\mathrm{NG}}-E_{\mathrm{Fe}}$$

where *E*_NG+Fe_, *E*_NG_, and *E*_Fe_ represent the total energies of Fe@N-doped graphene, N-doped graphene without Fe, and Fe atom/ion, respectively.

In the CO_2_RR calculation, all reaction Gibbs energies were calculated at 298 K and 1 atm using rigid-rotor, harmonic oscillator, and ideal gas approximations and principles of statistical thermodynamics. Transition states (TSs) were checked to display one imaginary frequency.

For water environment, we employed the computational hydrogen electrode (CHE) method^52^ to evaluate the reaction mechanism of the electrochemical CO_2_RR. The CHE approach was chosen due to its computational efficiency and its ability to incorporate the effect of an applied potential in DFT calculations. This method has been widely used for studying electrochemical reactions and screening electrocatalytic materials.^53^ The CHE method enables the estimation of free energy changes under electrochemical conditions without the need to explicitly model solvated ions, which are challenging to describe within standard DFT calculations. Using the CHE framework, we computed the Gibbs free energies of key intermediates involved in CO_2_RR. The reaction free energy at a given applied potential was determined using the following equation:

3 $$\Delta G=G\left(P\right)-G\left(R\right)-\frac{G\left(H_{2}\right)}{2}+0.059\mathrm{pH}+\left|e\right|U_{\mathrm{SHE}}$$

where *G*(P), *G*(R), and *G*(H_2_) represent the Gibbs free energies of the products, reactants, and H_2_ molecule, respectively, pH was set to 7, and *U* denotes the applied potential versus the standard hydrogen electrode (SHE). The potential of −0.7 V was used since we considered the formation of CO molecule as a final product.^24^ Grand-canonical DFT performed by Brimley et al.^54^ showed that lower applied reducing potential facilitated the production of CO; however, CO desorption became more prohibitive and possibly underwent further conversion to hydrocarbon products.

The natural bond orbital (NBO) analysis^55^ was used to analyze the charges on individual atoms/ions and thus to estimate the charge transfer within the system.

## Results

To systematically investigate defect formation in graphene and NG, we explored a wide range of vacancy structures to develop a comprehensive understanding of their prevalence, shapes, and size-determining factors. Following this, we examined the incorporation of iron into both pristine and nitrogen-doped defective graphene. Finally, we assessed the catalytic activity of Fe@NG for CO_2_RR. To establish general stability trends and governing principles for vacancy formation, we optimized a large data set of approximately 150 structures, encompassing vacancies of varying sizes (ranging from one to seven missing carbon atoms) and diverse shapes (Figures S4–S10). Our results for double and triple vacancies indicate that the formation of larger, more compact vacancies is energetically preferred over the presence of multiple smaller vacancies (Figure S5 and S6), aligning with previously reported findings.^56,57^ Consequently, our study primarily focuses on compact configurations of larger vacancies (Figures S7–S10) to better understand their structural stability and potential role in catalytic applications.

### Graphene Defects

First, we investigated the formation energies (eq 1) of defects in graphene (without any doping). These values varied significantly, ranging from 5.4 to 22.4 eV, with only a small subset of defects exhibiting *E*_form_ below 10 eV (SW­(55-77), SV, DV­(5-8-5), DV­(555-777), and DV­(5555-6-7777), Figures 1i, S1, and S4). This suggests that only a few vacancy types are likely to occur in the graphene sample at room temperature. At elevated temperatures, the number of probable vacancy types increases, as indicated by the Boltzmann distribution (Figure S11, eqs S1 and S2). The formation energy of a vacancy partially correlates with its size (Figure 1i,j). While this correlation is relatively weak when considering all computed structures (adjusted *R* ^2^ = 0.351, Figure 1i), it becomes significantly stronger withing the subset of the most stable vacancies of a given size (*R* ^2^ = 0.913, Figure 1j). This indicates that the vacancy stability generally decreases as more carbon atoms are removed from the graphene lattice.

**Figure 1:**
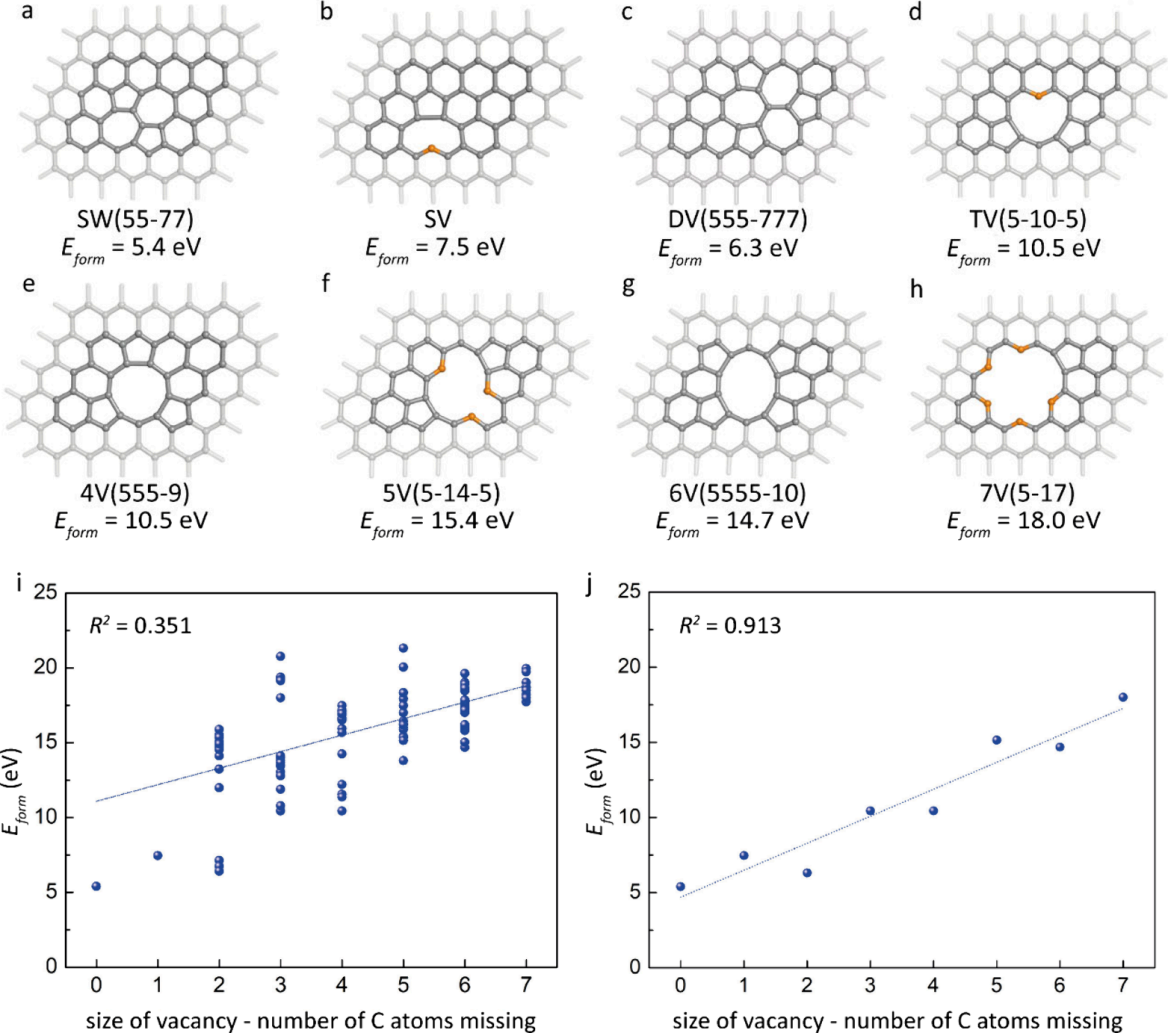
(a–h) Structures of the most stable vacancies for each vacancy size, ranging from zero to seven missing carbon atoms in graphene. Carbon atoms with dangling bonds are marked in orange. (i, j) Pair correlation between vacancy size and stability, shown as the relationship between formation energy (*E*_form_) and the number of missing carbon atoms in (i) all computed structures and (j) the most stable structures for each stoichiometry.

The Stone–Wales defect (SW­(55-77)) (Figure 1a, *E*_form_ = 5.4 eV) is the most stable among all considered structures, in agreement with previous DFT studies.^58,59^ It is followed by the double vacancy DV­(5-8-5) (*E*_form_ = 6.9 eV) and its two derivatives DV­(555-777) (*E*_form_ = 6.3 eV) and DV­(5555-6-7777) (*E*_form_ = 6.5 eV), formed by rotating one or two C–C bonds by 90° (Figures 1c and S1). The single vacancy (SV) (Figure 1b) is less stable due to the presence of a dangling bond. Notably, these *E*_form_ values align with previously reported DFT and experimental results.^40,60^ In general, vacancies with an odd number of missing adjoining carbon atoms prevent full C–C bond reconstruction, leaving dangling bonds in the structure (Figure 1). In contrast, vacancies with an even number of missing carbon atoms enable complete bond reconstruction, leading to higher stability, as observed in carbon nanotube studies.^61^ This trend is further supported by the significantly lower stability of other calculated DVs, which can be considered combinations of two SVs (Figure S5d–o), highlighting the destabilizing effect of the dangling bonds.

### Defects in Nitrogen Doped Graphene

Second, to investigate the stability of vacancies in NG, we selected 10 most stable vacancies in undoped graphene (Figure S12) and systematically replaced carbon atoms at the vacancy edge with nitrogen, varying from a single substitution to full replacement. The pair correlation between the number of edge N atoms and *E*_form_ (Figure 2a, b) shows that the incorporation of nitrogen(s) generally stabilizes the vacancies, which is consistent with previous findings.^62^ This trend is evident in both the full set of computed structures (Figure 2a) and the most stable vacancies for each stoichiometry (Figure 2b). The nitrogen’s different electron configuration enables complete bond reconstruction in the SV-1N structure, eliminating dangling bonds and stabilizing the vacancy compared to the pristine SV (*E*_form_[SV] = 7.5 eV, *E*_form_[SV-1N] = 5.2 eV, Figure 2c,d). Surprisingly, further substitution of an edge carbon in SV-1N with nitrogen enhances stability despite introducing a dangling bond in SV-2N (*E*_form_[SV-2N] = 4.8 eV, Figure 2e). However, the stabilization effect is weaker (Δ*E*_form_ = 0.4 eV for SV-2N vs SV-1N) compared to the initial nitrogen substitution (Δ*E*_form_ = 2.3 eV for SV-1N vs SV). This suggests that while the dangling bond in SV-2N contributes to destabilization, the reduced graphene lattice deformation (Figure 2d,e) counterbalances this effect. Both SV and SV-1N undergo Jahn–Teller distortion which helps saturate two dangling bonds and deforms the graphene lattice to form a pentagonal carbon ring. In contrast, SV-2N does not undergo Jahn–Teller distortion, leading to lower lattice deformation and increased stability. Additional nitrogen substitution (of carbon at the edge of SV-2N) further stabilizes the structure, with SV-3N exhibiting no dangling bond and no Jahn–Teller distortion, resulting in high stability (*E*_form_ = 3.4 eV, Figure 2f). A similar stability trend is observed for DV­(5-8-5) with varying numbers of nitrogen substitutions at the vacancy edge (Figure S13). However, unlike in SV-xN, the initial replacement of a carbon atom with nitrogen leads to vacancy destabilization (*E*_form_[DV­(5-8-5)] = 6.7 eV, *E*_form_[DV­(5-8-5)-1N] = 7.5 eV, Figure S13a,b). This destabilization arises from the introduction of a new dangling bond and the resistance of graphene lattice deformation caused by Jahn–Teller distortion. Subsequent nitrogen substitutions, however, stabilize the vacancy by saturating dangling bonds and reducing lattice deformation, similar to the trend observed in SV-xN (Figure S13c–e).

**Figure 2:**
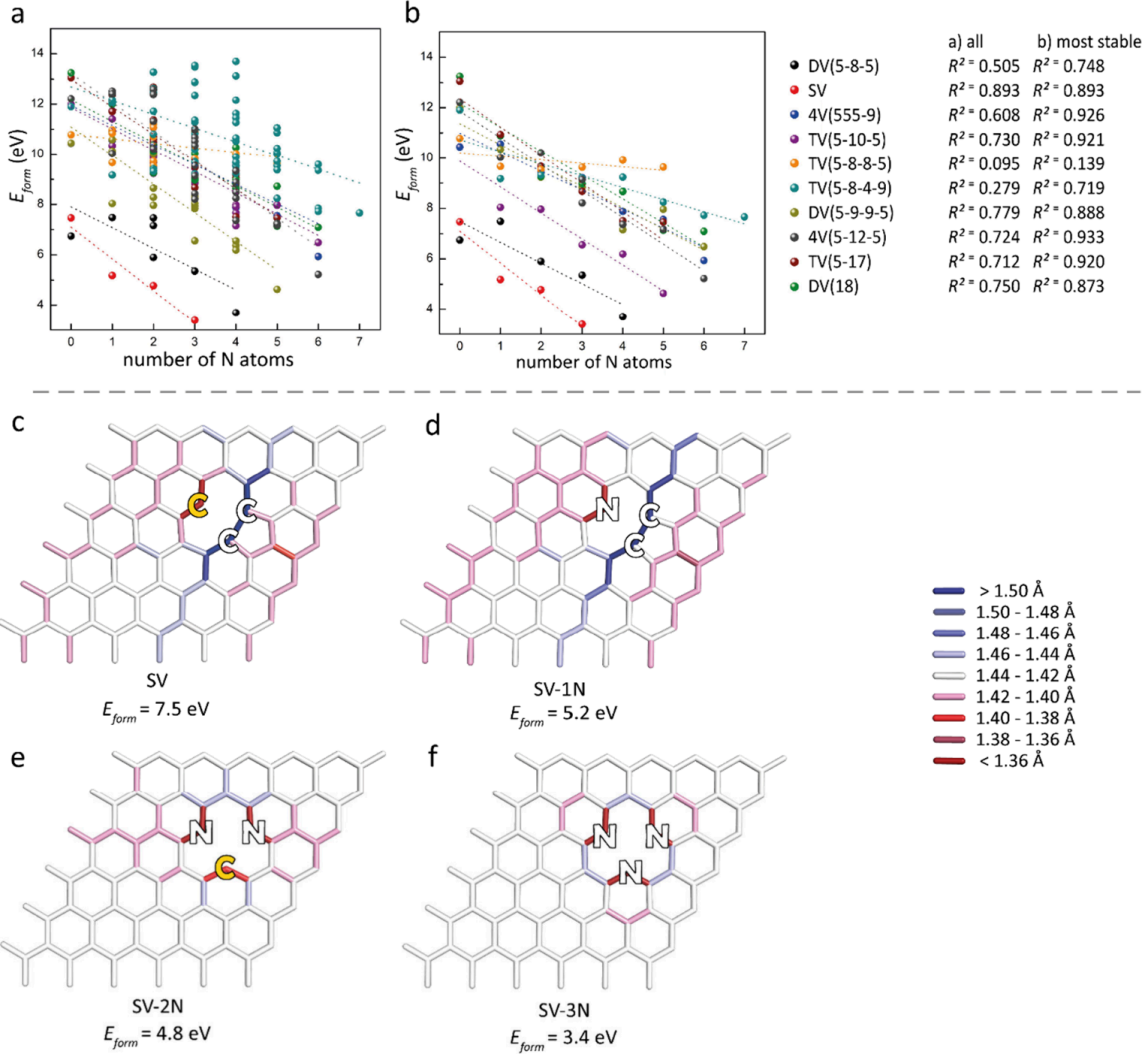
(a, b) Stabilization of vacancies in N-doped graphene through carbon-to-nitrogen substitution at vacancy edges demonstrated by the pair correlation between formation energy (*E*_form_) and the number of nitrogen atoms at the edges of (a) all computed N configurations and (b) the most thermodynamically favorable N positions. (c–f) The impact of dangling bonds (C atoms bearing dangling bonds are highlighted in orange) and graphene lattice deformation demonstrated by bond length variations, i.e., shortening and elongation of the C–C and C–N bonds relative to the pristine graphene C–C bond length (1.43 Å).

Nitrogen incorporation into the graphene lattice predominantly occurs at vacancy sites, leading to either complete or partial vacancy healing, consistent with previous studies.^30,63–65^ However, our finding reveals that the extent of healing strongly depends on vacancy size. For SVs, nitrogen incorporation is thermodynamically favored, resulting in the formation of graphitic nitrogen, full vacancy healing, and complete saturation of dangling bonds (Figure 3a). Similarly, for double vacancies (DV­(5-8-5)), full nitrogen-driven reconstruction remains energetically preferred (Figure 3b). In contrast, larger vacancies, such as triple vacancies (TV­(5-10-5)) and quadruple vacancies (4V­(555-9)) exhibit only partial healing. Nitrogen incorporation stabilizes these structures up to a certain point, but the process preferentially stops at intermediate stages, forming SV-2N and SV-3N, where two or three nitrogen atoms occupy edge sites (Figure 3c,d). This contrasts with the complete vacancy healing observed in the smaller defects. For even larger vacancies, nitrogen incorporation primarily stabilizes nitrogen-decorated vacancy edges rather than drives full reconstruction. A notable example is nitrogen incorporation into the hexavacancy 6V­(5555-10), which does not fully heal but instead transforms into a nitrogen-stabilized DV­(5-8-5)-4N structure (Figure S14a). These findings suggest that while nitrogen incorporation aids vacancy stabilization, its stabilizing effect is highly dependent on the vacancy size and structure.

**Figure 3:**
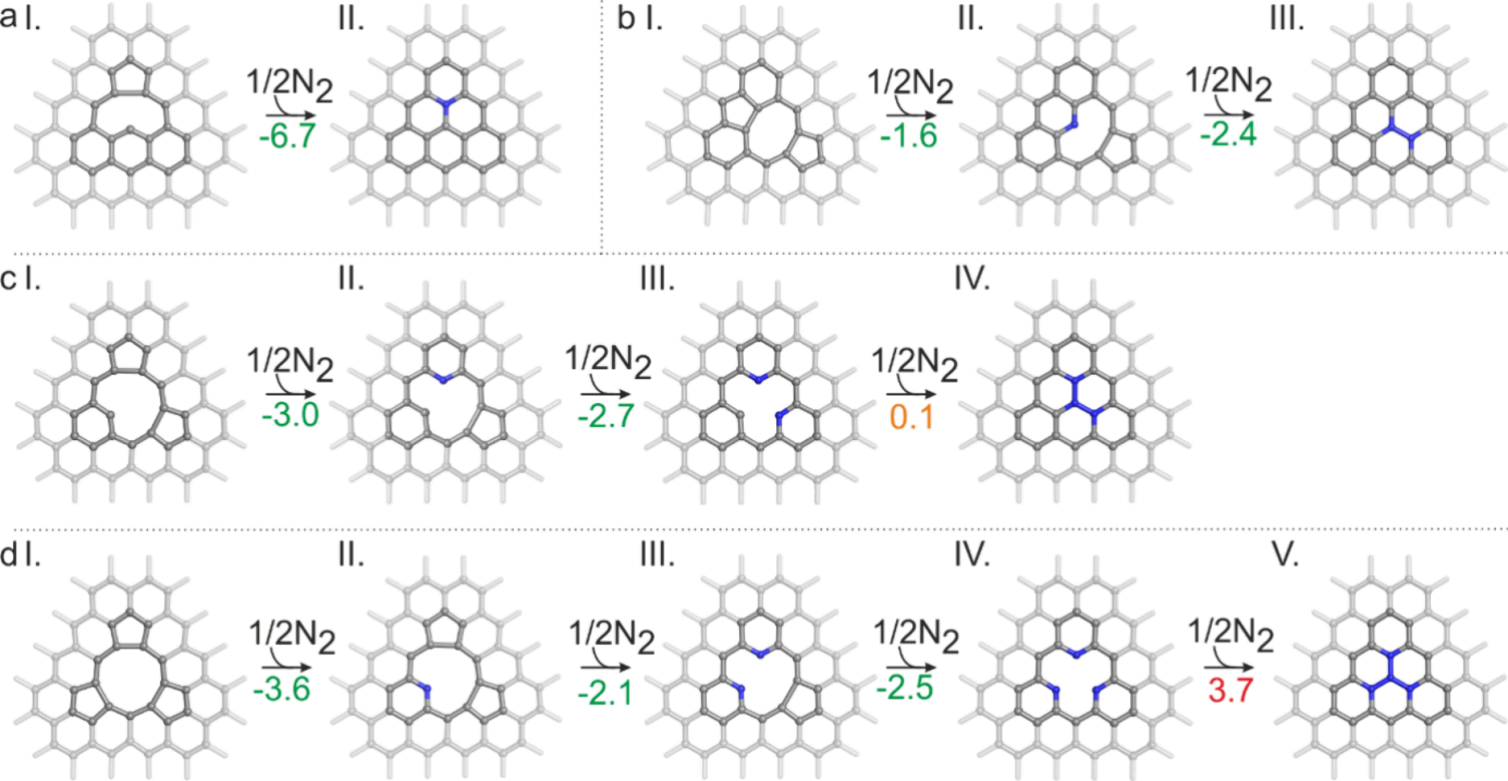
Step by step incorporation of nitrogen atoms into (a) SV, (b) DV­(5-8-5), (c) TV­(5-10-5), and (d) 4V­(555-9) resulting in either complete vacancy healing or the formation of smaller nitrogen-stabilized vacancies with nitrogen atoms at the edges. Reaction energies (Δ*E*_R_) are displayed in eV. Carbon atoms are shown in gray, and nitrogen atoms are shown in blue.

Overall, the significantly higher stability of SW­(55-77), SV, DV­(555-777), DV­(5-8-5), and DV­(5555-6-7777) among other calculated vacancies indicates that these defect types will be the most prevalent in real graphene samples. Nitrogen doping is expected to induce complete or partial healing of these vacancies, leading to the formation of graphitic nitrogen and SV-1N (Figure 3a,b). However, lager vacancies such as TV­(5-10-5), 4V­(555-9), and 6V­(5555-10) (Figures S6a, S7a, and S9a) can be introduced into the graphene lattice in a controllable manner using electron or ion irradiation.^66^ The healing of these larger vacancies through nitrogen incorporation leads to the formation of nitrogen-stabilized defects such as SV-2N (Figure 3c-III), SV-3N (Figure 3d-IV), and DV­(5-8-5)-4N (Figure S14a). The stabilities of structures such as 4V­(5-5-5-9)-6N, TV­(5-10-5)-5N, TV­(5-8-8-5)-5N, TV­(5-8-4-9)-7N, DV­(5-9-9-5)-6N, 4V­(5-12-5)-6N, TV­(5-17)-5N, and DV(18)-6N (Figure S12) further suggest that nitrogen doping in highly defective graphene could stabilize vacancies as large as 8V (where eight carbon atoms are removed) or even larger, making their presence feasible in real samples.

### Iron Embedding into Defective Nitrogen-Doped Graphene

SACs based on iron embedded in NG lattice can be experimentally prepared by soaking of iron (II/III) salts with NG in water.^67^ Subsequently, they can be treated, e.g., reduced in the gas phase, for CO_2_RR catalysis.^18,68^ Following this concept and the above-established preferences for vacancy formation, we investigated vacancies as potential anchoring sites for Fe single atomic species. Given that single vacancies (Fe@(N)­SV) and double vacancies (Fe@(N)­DV) were among the commonly occurring defects in graphene and NG, our focus was primarily on these structures. We further altered the number and the positions of N atoms in the closest vicinity of Fe, i.e., the nearest nitrogen atoms (NN) surrounding Fe (Figure 4). Analysis of interaction energies between Fe and NG unraveled that the interaction strongly depends on the Fe/N ratio and Fe/N arrangement, charge, multiplicity, and solvent (Figure 4 and Tables S2–S6). We considered embedding of Fe^3+^/Fe^2+^ ions in the water phase and of Fe^3+^/Fe^2+^/Fe^0^ species in the gas phase. To explore the effect of multiplicity, we first identified the most favorable multiplicity of isolated Fe in different charge states and then varied the multiplicity upon embedding in Fe@NG to assess its impact on the binding based on interaction energies. Even though no clear preference for a specific spin state was revealed for the studied Fe@NG systems, the multiplicity of 3–6 was preferred over lower or higher multiplicities (Tables S2–S6).

**Figure 4:**
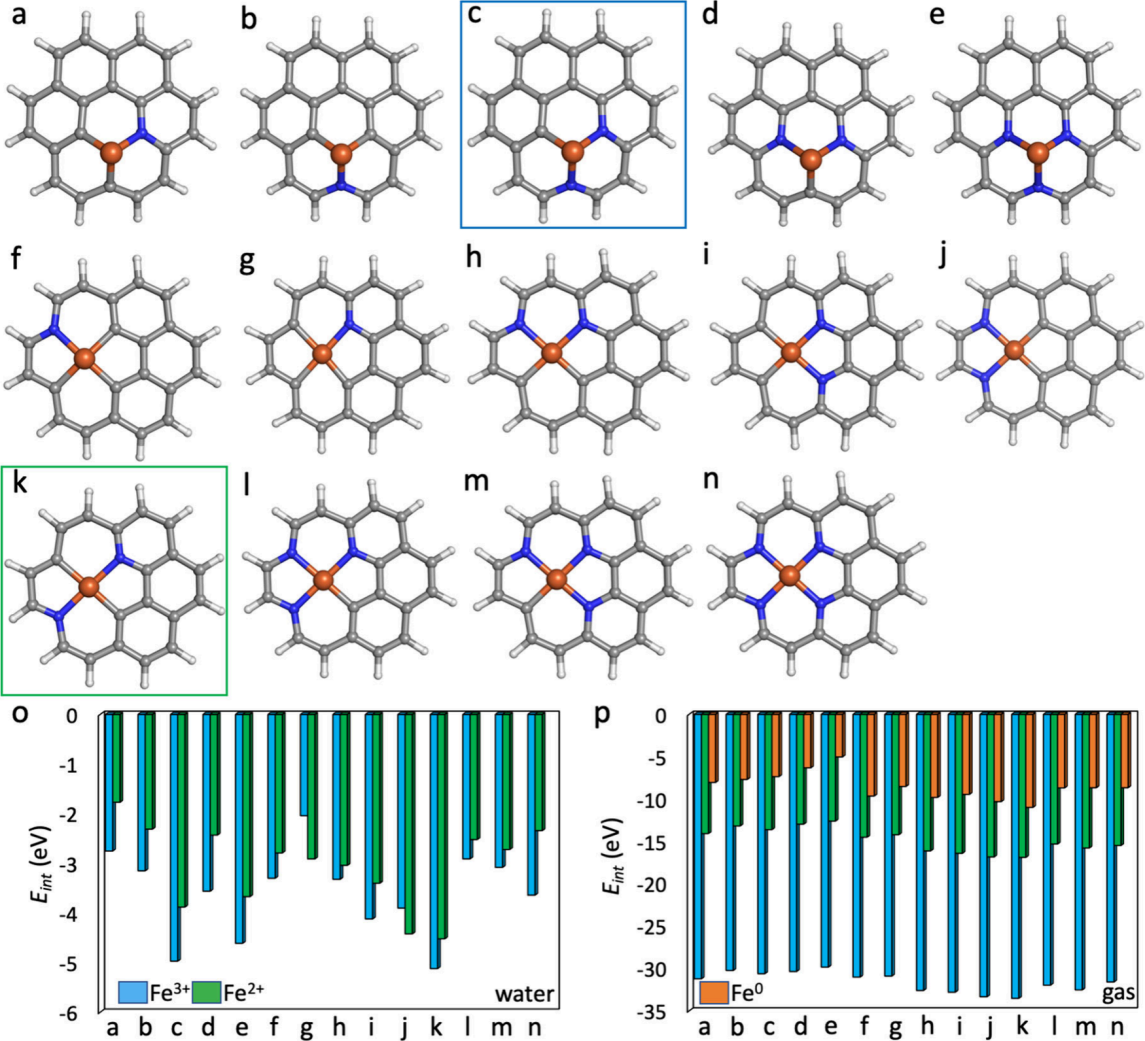
Coronene models of Fe@NG with NN atoms. Structures correspond to Tables S2–S6. Carbon in gray, nitrogen in blue, iron in ochre, and hydrogen in white. Blue/green squares depict the most stable arrangement of Fe@NSV and Fe@NDV, respectively. Energy diagrams showing the interaction energies of Fe@NG with NN atoms: (o) water solvent; (p) gas phase. The most favorable multiplicity states are depicted. The corresponding energies are shown in Tables S2–S6.

Fe ions showed lower interaction energies to NG in the gas phase than equivalent systems in water. This can be explained by a significant role of the electrostatic interaction, which is screened in water. In the gas phase, the absence of solvent screening results in stronger electrostatic interactions between the Fe ions and the NG support. These interactions are screened by the solvent as the dielectric constant of water reduces the electrostatic interactions compared to the gas phase. In water, Fe­(III)@NG displayed lower interaction energies (from −0.56 to −5.12 eV) than Fe­(II)@NG (from 0.24 to −4.52 eV) in the respective systems. This suggests that binding of Fe^3+^ is preferred in water phase than biding of Fe^2+^. It is also worth noting that there is a significant charge transfer from NG to Fe^II/III^ as indicated by the NBO analysis. Unlike an isolated Fe^3+^ or Fe^2+^ ion, Fe in NG did not exhibit a full 3+ or 2+ oxidation state (Figure S15). Instead, partial electron retention in the 3d and 4s/4p orbitals suggests charge delocalization across Fe and NG, likely due to strong metal–support interactions and charge back-donation effects mainly to 3d and 4s orbitals. In the gas phase, we also considered the presence of an Fe^0^ atom embedded in the respective active sites, because it can be prepared by reduction of Fe^II/III^ containing systems. Fe­(III)@NG was the most strongly bound to NG in the gas phase (from −28.51 to −33.52 eV), then Fe­(II)@NG (from −10.34 to −16.88 eV), and Fe(0)@NG (from −3.13 to −10.98 eV). The NBO analysis revealed that Fe^0^ in NG exhibited an electron depletion of 4*s* electrons and an increase in 3d and 4p occupancy. While Fe was not fully oxidized, it carried a slight partial positive charge (Figure S15).

Based on the interaction energies, Fe preferred to reside in the DV surrounded by two nitrogen atoms and two carbon atoms arranged in a diagonal manner (Fe@C_2_N_2_) as shown in Figure 4k. As for Fe@NSV, Fe was preferentially bound to two nitrogen atoms and one carbon atom (Fe@C_1_N_2_, Figure 4c). The results were consistent for almost all Fe@NDV variants, regardless of charge and solvent, only Fe(0)@NSV in the gas phase showed Fe@C_2_N_1_ (Figure 4a) as the most preferred Fe@NSV. Since the most stable Fe–N arrangements reported in the literature showed ambiguity across different setups,^25,69,70^ we assessed the quality of the method and basis set by performing calculations using three distinct computational configurations, as outlined in the Computational Details section. All three computational methods revealed the same most stable Fe–N arrangements, confirming the consistency and reliability of the results across different setups (Table S7). Furthermore, we considered a larger model of graphene, circumcoronene (Figure S16), to validate the trends observed for smaller coronene models of Fe@NG. Across the considered charges and solvents, the most preferred Fe embedding to NSV and NDV remained unchanged besides Fe(0)@NSV in the gas phase showing the Fe@C_1_N_2_ pattern as the most stable, thus eliminating the above-mentioned discrepancy of the coronene model (Tables S8–S12). It is also worth noting that formation of the Fe@C_2_N_2_ pattern appeared to be thermodynamically feasible (Figure S14b), took less steps than formation of the Fe@N_4_ and started from more probable vacancy (4V­(5-12-5), *E*_form_ = 12.2 eV, Figure S7c) than in the case of Fe@N_4_ (6V­(5555-10), *E*_form_ = 14.7 eV, Figure S9a).

Beyond the commonly studied NN atoms around Fe,^24,71,72^ we also investigated the next-nearest nitrogen atoms (NNN), distant nitrogen atoms (DN) and edge nitrogen atoms bonded to hydrogen, using both smaller coronene (Figures S17 and S18) and larger circumcoronene models (Figure S19). Given the numerous possible structural arrangements, we limited calculations to systems with a multiplicity of 3–6, as these appeared to be preferred. Notably, most Fe@NSV and Fe@NDV structures with NNN and DN configurations were found to possess interaction energies lower than those in which Fe was surrounded solely by NN atoms (Tables S13–S16). For both coronene and circumcoronene models, Fe@NDV, shown in Figure 5r, and Figure S19q/r, exhibited the lowest interaction energies among all considered structures, where nitrogen atoms were positioned in neighboring hexagonal rings in a para configuration (Tables S13–S16). As for Fe@NSV, nitrogen atoms and Fe were preferentially located in a hexagon of graphene (Figure 5h and Figure S19i). The stability trend in terms of Fe charge and solvent remained unchanged as compared to Fe@NG surrounded only by NN, i.e., in water solvent Fe^3+^ > Fe^2+^ and in gas phase Fe^3+^ > Fe^2+^ > Fe^0^ (Tables S13–S16).

**Figure 5:**
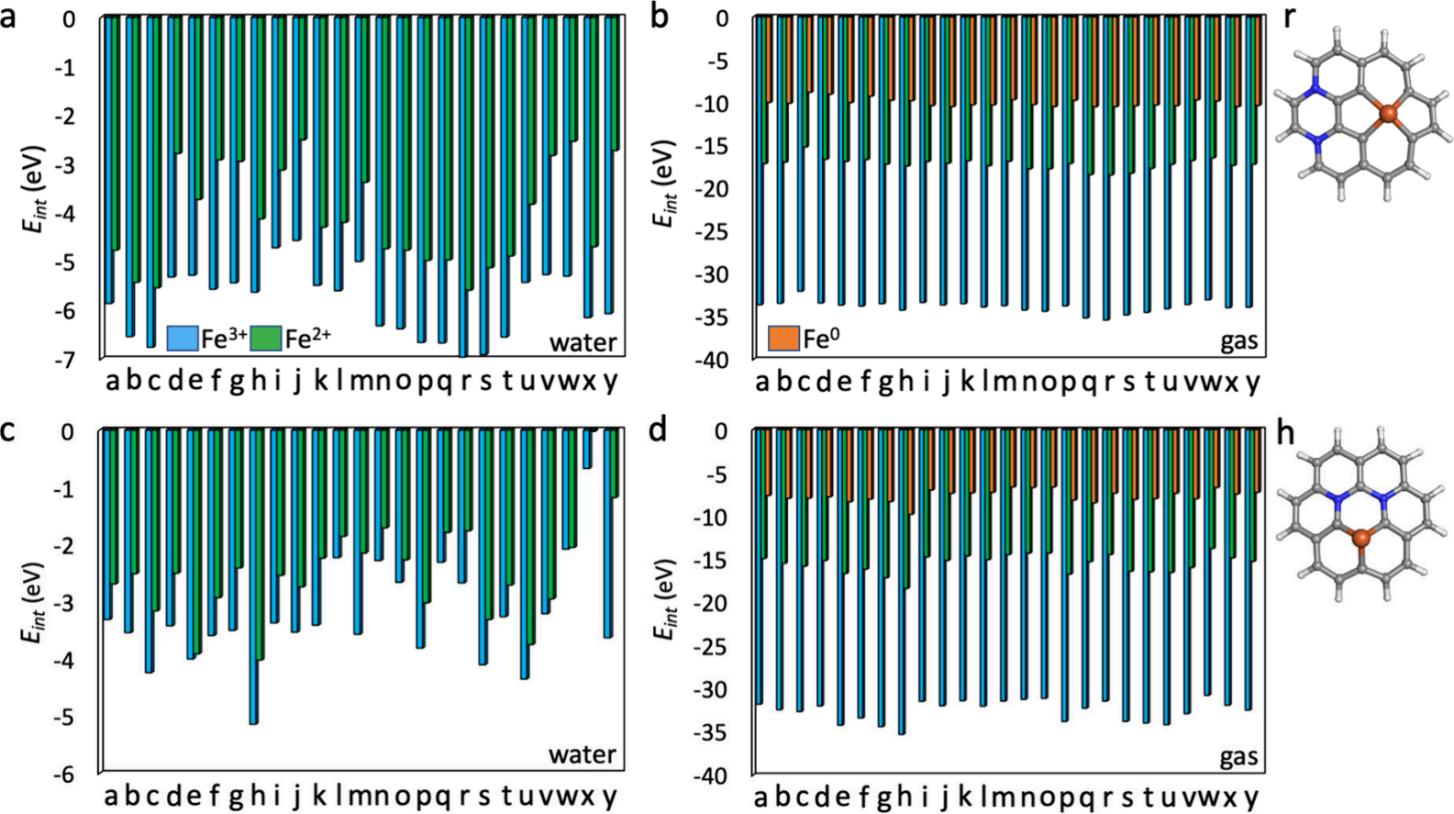
Energy diagram showing the interaction energies of Fe@NG with NNN atoms: (a) Fe@NDV, water solvent, (b) Fe@NDV, gas phase, (c) Fe@NSV, water solvent, (d) Fe@NSV, gas phase. (r) The most stable Fe–N arrangement of Fe@NDV and (h) the most stable Fe–N arrangement of Fe@NSV. The most stable multiplicity states are depicted. The corresponding structures and energies are shown in Figures S17 and S18 and Tables S13 and S14.

### Catalytic Activity of Iron Containing Nitrogen-Doped Graphene

While structural stability is often a key objective in catalyst design, it does not necessarily correlate with high catalytic performance.^73–75^ A critical yet often overlooked question is whether the most stable structure characterized by the lowest energy is also the most active, e.g., offering optimal (minimum energy) reaction pathways or if there is an inherent trade-off between stability and activity. To address this, we analyzed the binding preferences of iron in NG and assessed its catalytic efficiency in terms of energy profiles along reaction intermediates of CO_2_RR. We focused on three key iron binding configurations in NG (Figure 6): Fe@C_2_N_2_ pattern, identified as the most preferred Fe–N composition in Fe@NDV with NN atoms, the Fe@N_4_ pattern, commonly used in catalytic reactions,^24,76,77^ and the most stable configuration of Fe@ND­(S)­V. We then investigated the catalytic activity of these structures for the carbon dioxide reduction reaction (CO_2_RR) (Figure 6),^31,78^ considering various charge states and multiplicities of iron (Tables S17–S21).

**Figure 6:**
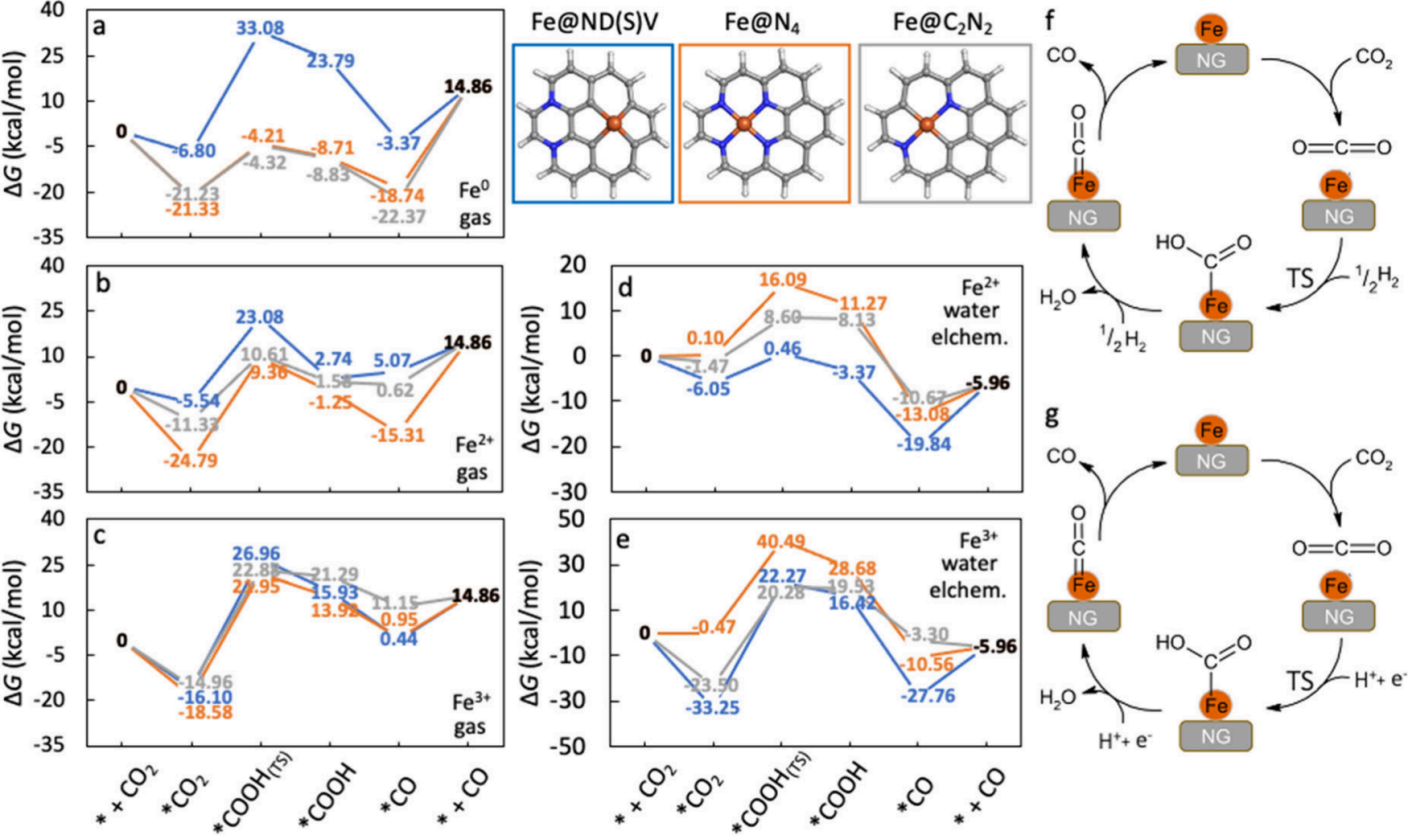
CO_2_RR catalyzed by Fe@NG. (a) Fe(0)@NG in gas phase, (b) Fe­(II)@NG in gas phase, (c) Fe­(III)@NG in gas phase, (d) Fe­(II)@NG in water solvent, (e) Fe­(III)@NG in water solvent, (f) scheme of the CO_2_RR path catalyzed by selected Fe@NG in the gas phase, and (g) scheme of the electrochemical CO_2_RR path catalyzed by selected Fe@NG in water solvent. The energy profiles in blue, gray, and orange correspond to the systems highlighted by the frames of the same colors.

Focusing on the CO_2_RR in the gas phase, the reaction profile was significantly influenced by both the oxidation state of iron and the arrangement of active sites. In all cases, the adsorption of CO_2_ is thermodynamically favorable. For the zerovalent iron embedded in NG (Figure 6a), the thermodynamically most stable catalyst Fe@ND­(S)V exhibited the highest transition state (TS) barrier (39.9 kcal/mol) associated with formation of −COOH, making it the most energetically demanding step along this reaction profile. The other two systems, i.e., Fe@N_4_ and Fe@C_2_N_2_, significantly reduced the barriers for the TS formation to 17.1 and 16.9 kcal/mol, respectively, at the expense of higher energy required for CO desorption, which became the limiting process with required Gibbs energies of 33.6 and 37.2 kcal/mol, respectively. The less thermodynamically stable catalysts may provide more efficient reaction pathway with different rate limiting processes.

In the case of Fe^II^ (Figure 6b), the pathway catalyzed by Fe@N_4_ displayed the barrier for formation of the −COOH intermediate (34.1 kcal/mol) as the most energetically demanding step, as the CO desorption required 30.2 kcal/mol. The other two systems, Fe@C_2_N_2_ and Fe@ND­(S)­V, had lower energy gains associated with the adsorption of CO_2_ and lower energies needed for CO desorption with respect to Fe@N_4_. Fe@C_2_N_2_ efficiently stabilized the TS with an associated barrier of 21.9 kcal/mol and rendered itself as the most efficient catalyst with the rate limiting step associated with formation of −COOH intermediate. It was followed by Fe@ND­(S)V with barrier of 28.6 kcal/mol. For Fe^III^ (Figure 6c), the formation of the TS limited the catalytic process in all cases with associated barriers of 37.8, 40.5, and 43.1 kcal/mol, for Fe@C_2_N_2_, Fe@N_4_, and Fe@ND­(S)­V, respectively.

For the water environment, we employed the computational hydrogen electrode (CHE) method to evaluate the reaction mechanism of the electrochemical CO_2_RR. The reaction profile in water solvent was significantly influenced by both the oxidation state of Fe and the nature of the active site. Concerning Fe^II^ (Figure 6d), Fe@ND­(S)V showed the most favorable adsorption energy for CO_2_ and the lowest energy barrier of the TS (6.5 kcal/mol). In this case, desorption of the CO molecule (13.9 kcal/mol) represented the limiting step. In contrast, Fe@N_4_ and Fe@C_2_N_2_ had the TS formation as the limiting step, with barriers of 16.0 and 10.1 kcal/mol, respectively. The CO desorption required 7.1 and 4.7 kcal/mol for Fe@N_4_ and Fe@C_2_N_2_, respectively. So Fe@C_2_N_2_, represented the most efficient electrocatalyst among considered systems. For Fe^III^ (Figure 6e), the formation of TS was prohibitively expensive with barriers ranging from 41.0 kcal/mol (Fe@N_4_) to 55.5 kcal/mol (Fe@ND­(S)­V).

The NBO analysis of Fe@NG in both gas and water solvent revealed that, as compared to the freestanding Fe@NG substrates, the Fe charge decreased upon the adsorption of CO_2_ and subsequent intermediates due to electron donation to the 3d and 4s orbitals of Fe from the adsorbed molecules. Notably, the amount of transferred charge, together with the varying adsorption energies of CO_2_, indicated that both physisorbed and chemisorbed states occurred,^79,80^ depending on the Fe@NG substrate, charge, multiplicity, and solvent.

These results clearly demonstrate that the active sites of the catalyst significantly influence the catalytic pathway. They also reveal that the most stable catalytic sites are not necessarily the most efficient, highlighting a trade-off between catalyst stability (and thus reusability) and catalytic performance. However, our study considered only one reaction, one reaction mechanism, and three binding modes of a single transition metal. Therefore, future studies should explore a broader set of catalytic reactions and active site configurations to establish fundamental principles governing the efficiency of single-atom catalysts (SACs).

## Conclusion

This study systematically investigated the stability of vacancies in undoped graphene and their implications for nitrogen doping and iron binding. Our analysis determined that only a few types of vacancies are likely to occur and that vacancies displaying an even number of absent carbon atoms tend to maintain higher stability, attributed to complete bond reconstruction. The stability of vacancies decreases with increasing vacancy size, and vacancies with an even number of missing carbon atoms exhibit higher stability due to full bond reconstruction. Our results confirm that larger, compact vacancies are more stable than multiple smaller ones. The incorporation of nitrogen at the edges of the most stable vacancies stabilizes the vacancies. In addition, nitrogen doping can effectively eliminate smaller vacancies, transforming them to nitrogen doped graphene, while larger ones remain open, albeit stabilized. Iron can bind to defective NG. Moreover, the interaction of iron with NG demonstrates that the charge state, spin state, and solvent environment influence the stability and functional properties of the Fe@NG structures. In addition, the same properties significantly modulate energies along CO_2_RR pathway. Importantly, our analysis of the CO_2_RR on various Fe@NGs indicated that the most thermodynamically stable active site does not necessarily enable the energetically most favorable profile and hence the highest catalytic activity. Instead, kinetically accessible yet less stable Fe–N motifs may offer superior catalytic performance. These findings highlight the importance of understanding the interplay between the vacancy type, the nitrogen concentration, and the local charge environment in optimizing the design of high-performance catalysts. Evaluating various binding modes (and oxidation states) of iron differing in their stability, we revealed a balance between catalyst stability and catalytic activity. Our work opens new doors for future research into vacancy engineering and the rational design of single-atom catalysts, with significant implications for catalytic reactions and energy conversion processes. Furthermore, incorporating a wider range of metals, exploring diverse catalytic reactions, and leveraging machine learning techniques could help to identify the fundamental principles of SAC activity and accelerate the development of more efficient and targeted SACs.

## Supplementary Material



## Notes

Formation energies obtained from parameter testing (Table S1); optimized models and formation energies of defective graphene supercells and finite models (Figures S1–S10); Boltzmann distribution and formation energies of defects in graphene (Figure S11); 10 most stable vacancies in undoped graphene and their counterparts (Figure S12); deformation of the graphene plane with various defects in the lattice (Figure S13); step by step incorporation of nitrogen atoms and an iron atom (Figure S14); NBO analysis of selected Fe@NG graphene with high Fe stability (Figure S15); interaction energies of Fe@NG (Tables S2–S16); optimized models of Fe@NG (Figures S16–S19); CO_2_RR catalyzed by Fe@NG (Tables S17–S21) (PDF)

## Abbreviations

NG: nitrogen-doped graphene
SACs: single-atom catalysts
Fe-SACs: iron-based SACs
DFT: density functional theory
SV: single vacancy
DV: double vacancy
TV: triple vacancy
4V: quadruple vacancy
5V: quintuple vacancy
6V: sextuple vacancy
7V: septuple vacancy
Fe@NG: iron embedded in nitrogen-doped graphene
ORR: oxygen reduction reaction
CO_2_RR: CO_2_ reduction reaction
TM: transition metal
PBC: periodic boundary conditions
SP-DFT: spin-polarized density functional theory
PBE: Perdew, Burke, and Ernzerhof
PAW: projected augmented wave potentials
VASP: Vienna ab initio simulation package
SMD: solvation model based on electron density
CHE: computational hydrogen electrode
NBO: natural bond orbital
