# Supplementary material for: Iron Single-Atom Catalysts Anchored on Defect-Engineered N‑Doped Graphene Reveal an Interplay between CO2 Reduction Activity and Stability
Source: ACS Sustain Chem Eng. 2025 May 28;13(22):8319–30. doi: 10.1021/acssuschemeng.5c01417 (PMC12153042; doi:10.1021/acssuschemeng.5c01417)
Supplement: Supplementary file 1 [file sc5c01417_si_001.pdf]

Iron Single-Atom Catalysts Anchored on Defect-Engineered N-Doped Graphene Reveal an Interplay Between CO_2 Reduction Activity and Stability

Dagmar Zaoralová [†#], Rostislav Langer [†#], Michal Otyepka [†‡*]

[†] IT4Innovations, VSB–Technical University of Ostrava, 17. listopadu 2172/15, 708 00 Ostrava-Poruba, Czech Republic.

[‡] Regional Centre of Advanced Technologies and Materials, The Czech Advanced Technology and Research Institute (CATRIN), Palacký University Olomouc, Šlechtitelů 27, 779 00 Olomouc, Czech Republic

# Equally contributed to this work

* Michal Otyepka. Michal.Otyepka@upol.cz

Number of pages: 37

Number of figures: 19

Number of tables: 21

Computational details

To evaluate the possibility of various defects in graphene we have calculated the Boltzmann distribution at 300 K, 600 K, and 1200 K (**Figure S11**) as follows:

$$p_i = \frac{e^{-\frac{E_{form,i}}{k_B T}}}{Q}, \qquad \text{(S1)},$$

where the partition function Q is expressed as:

$$Q = \sum_j \quad e^{-\frac{E_{form,j}}{k_B T}}, \qquad \text{(S2)},$$

where p_i is the probability of the *i-th* defect, E_{form} is its formation energy, the summation runs over all considered defects, k_B is the Boltzmann constant and T is the thermodynamic temperature (300 K, 600 K and 1200 K).

The reaction energies in **Figure S14** were calculated as follows:

$$\Delta E_R = E_{prod} - E_{react} - E_{Fe\ atom/\frac{1}{2}N_2}, \qquad \text{(S3)},$$

Where ΔE_R represents the reaction energy, E_{prod} is the total energy of the product of given reaction step, E_{react} is the total energy of the corresponding reactant, and $E_{Fe\ atom/\frac{1}{2}N_2}$ is the total energy of single Fe atom or half of the total energy of an N_2 molecule.

Table S1: The formation energies (E_{form}) obtained from parameter testing on DV(5-8-5)-4N defect (**Figure S12a**) which determined the used settings for the cut-off energy and the number of k-points (highlighted).

**Cut-off energy (eV)**	**E_{form} (eV)**		**k-points**	**E_{form} (eV)**
**300**	3.52		1x1x1	3.18
**400**	3.72		3x3x1	3.72
**500**	3.74		5x5x1	3.70
**600**	3.76		7x7x1	3.70
**700**	3.75		9x9x1	3.71

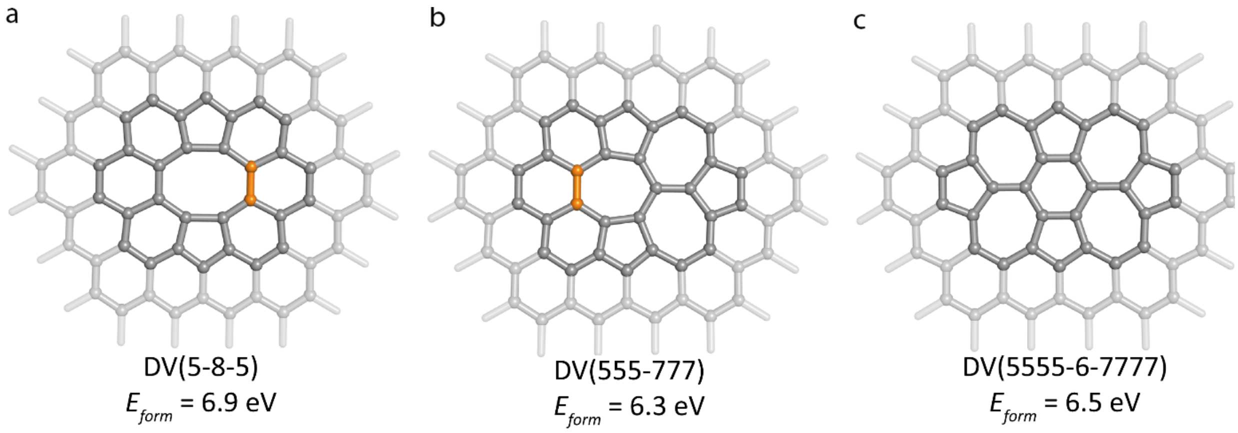

Figure S1: Structures of the most stable double vacancies and their formation energies (E_{form}). a) DV(5-8-5) that was created by removing two carbon atoms from the graphene lattice, b) DV(555-777) that was created by rotation by 90° of the orange marked C-C bond in DV(5-8-5), and c) DV(5555-6-7777) that was created by rotation by 90° of the orange marked C-C bond in DV(555-777). The carbon atoms around the vacancy are darker for better clarity.

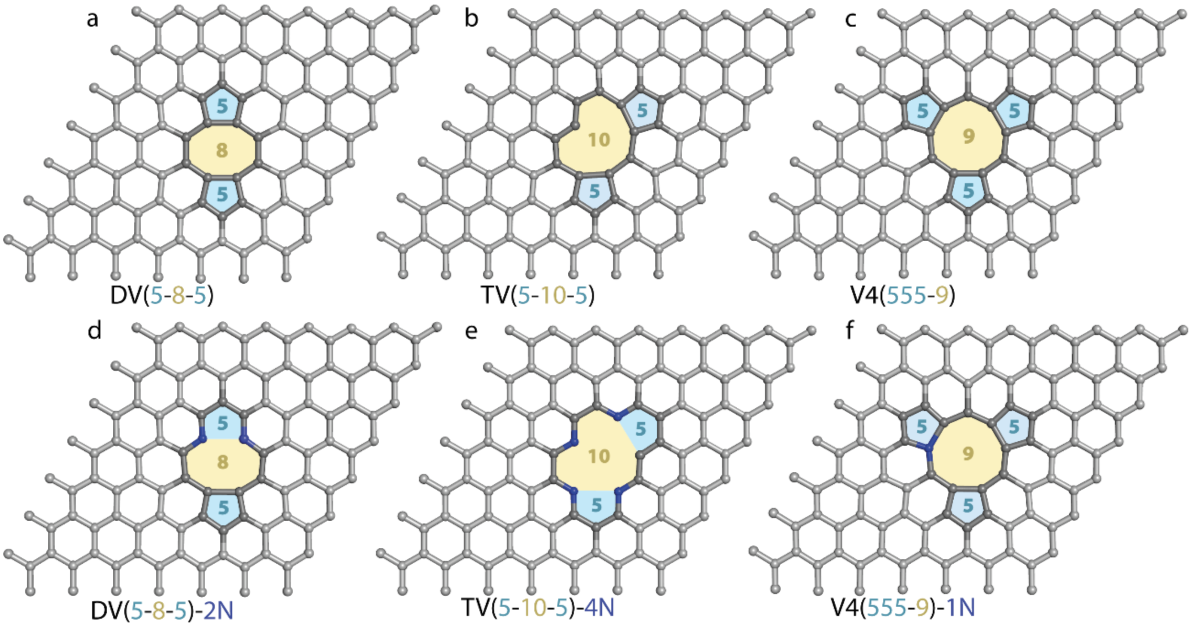

Figure S2: Schematic representation of defects in a-c) graphene and d-f) nitrogen-doped graphene. The labeling reflects the number of missing carbon atoms, with "DV" for a double vacancy, "TV" for a triple vacancy, and "V4" for a quadruple vacancy, corresponding to the removal of two, three, and four carbon atoms, respectively. The numbers in parentheses indicate the size of carbon/nitrogen atom rings formed after vacancy optimization. Last two characters in d-f) indicate number of nitrogen atoms on the edge of the vacancy. Carbon atoms are shown in grey, and nitrogen atoms in blue.

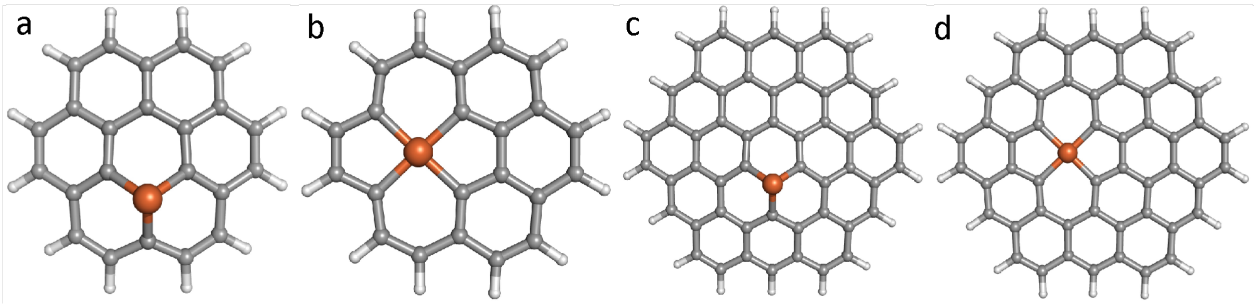

Figure S3: Coronene model of a) Fe@SV and b) Fe@DV. Circumcoronene model of c) Fe@SV and d) Fe@DV. Carbon atoms shown as grey spheres, iron in ochre, hydrogen in white.

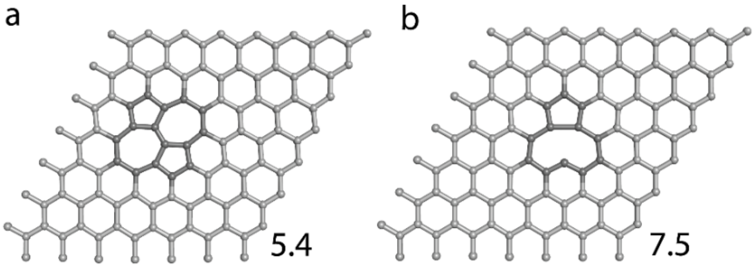

Figure S4: Structures of optimized defective graphene supercells with a) Stone-Wales (SW(55-77)) defect and b) single vacancy (one carbon atom is removed from the lattice of graphene). Numbers denote formation energies (E_{form}) in eV. The carbon atoms at the edge of the vacancy are darker for better clarity.

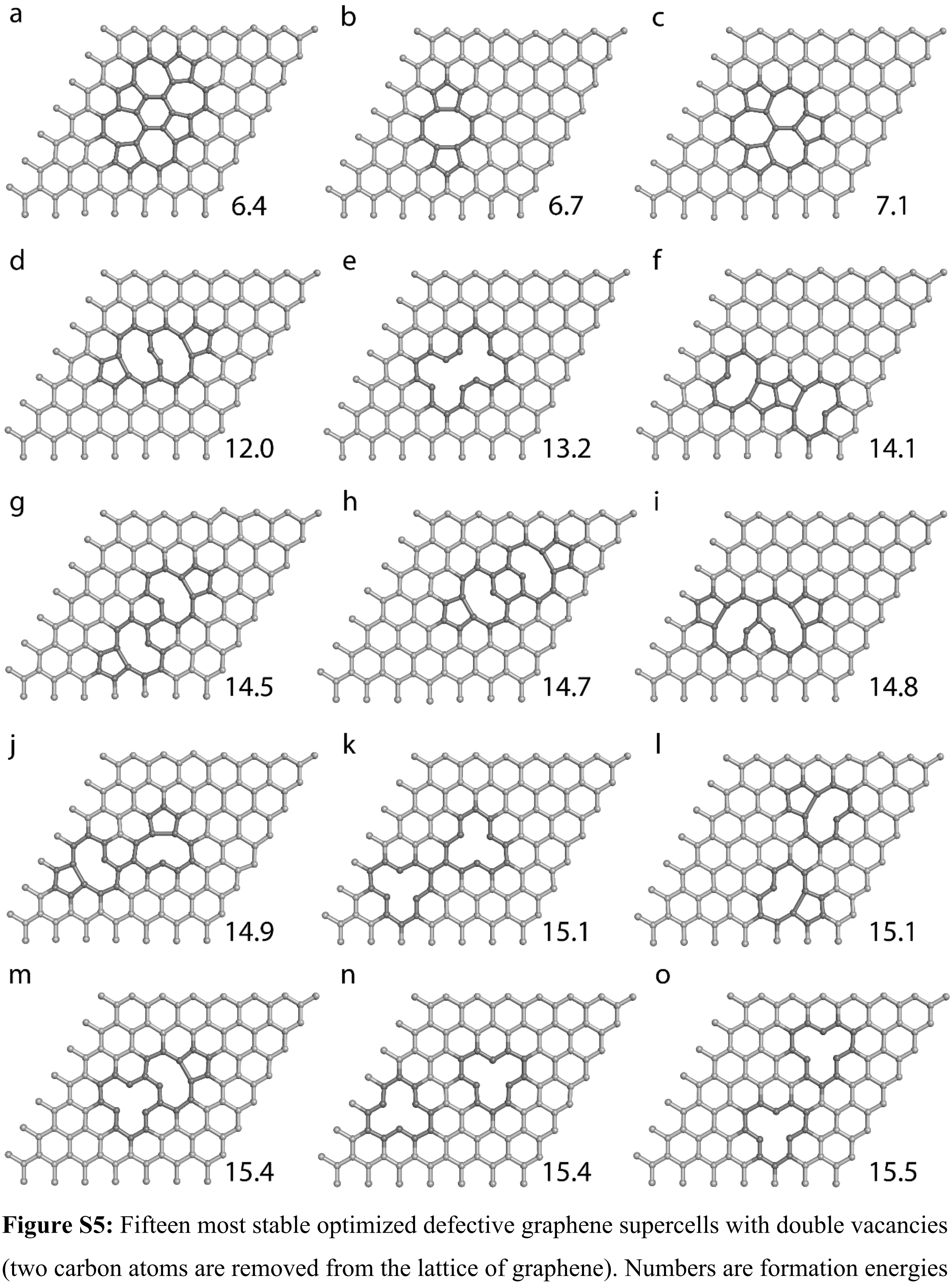

Figure S5: Fifteen most stable optimized defective graphene supercells with double vacancies (two carbon atoms are removed from the lattice of graphene). Numbers are formation energies (E_{form}) in eV. The carbon atoms at the edge of the vacancy are darker for better clarity.

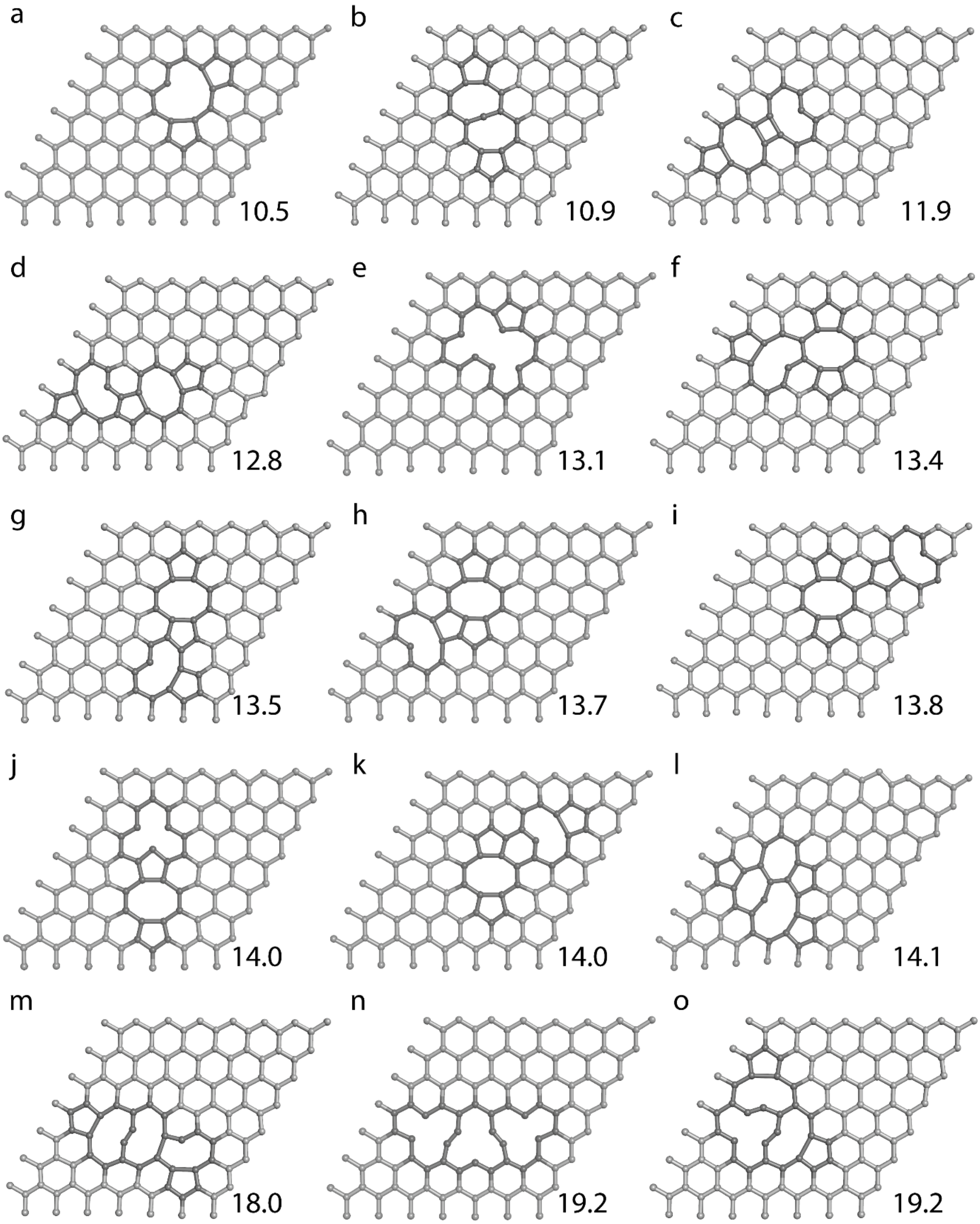

Figure S6: Fifteen most stable optimized defective graphene supercells with triple vacancies (three carbon atoms are removed from the lattice of graphene). Numbers are formation energies (E_{form}) in eV. The carbon atoms at the edge of the vacancy are darker for better clarity.

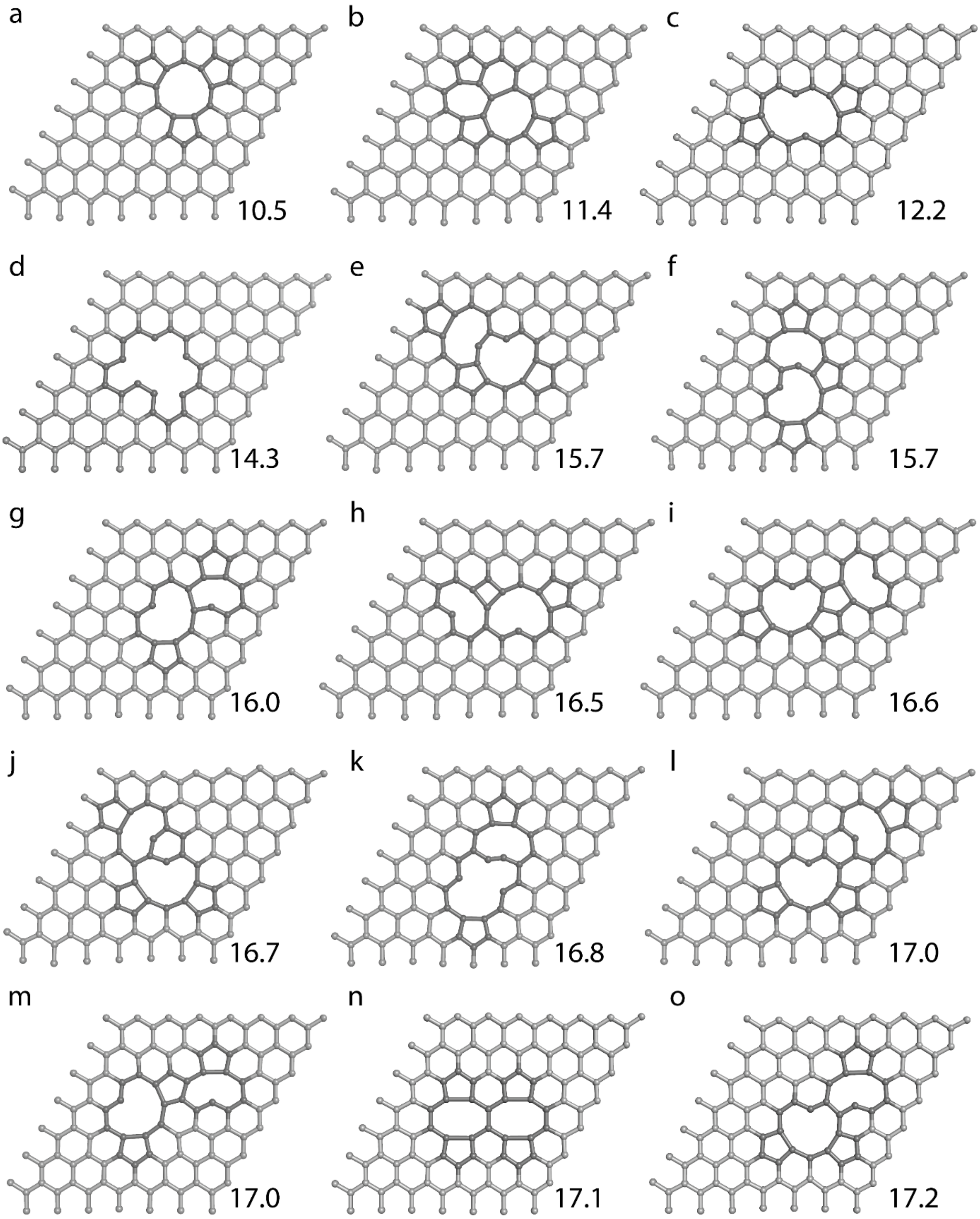

Figure S7: Fifteen most stable optimized defective graphene supercells with quadruple vacancies (four carbon atoms are removed from the lattice of graphene). Numbers are formation energies (E_{form}) in eV. The carbon atoms at the edge of the vacancy are darker for better clarity.

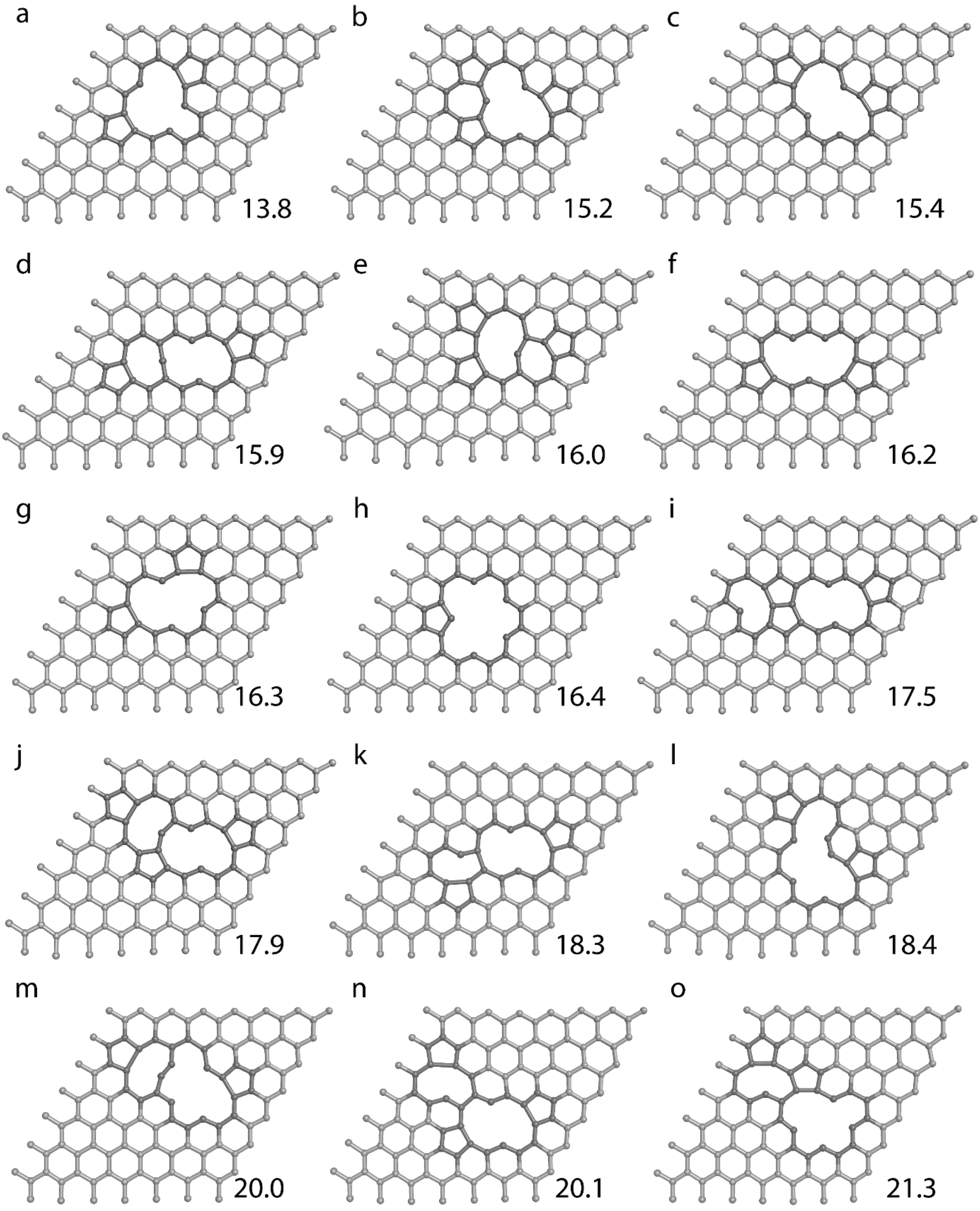

Figure S8: Fifteen most stable optimized defective graphene supercells with quintuple vacancies 5V (five carbon atoms are removed from the lattice of graphene). Numbers are formation energies (E_{form}) in eV. The carbon atoms at the edge of the vacancy are darker for better clarity.

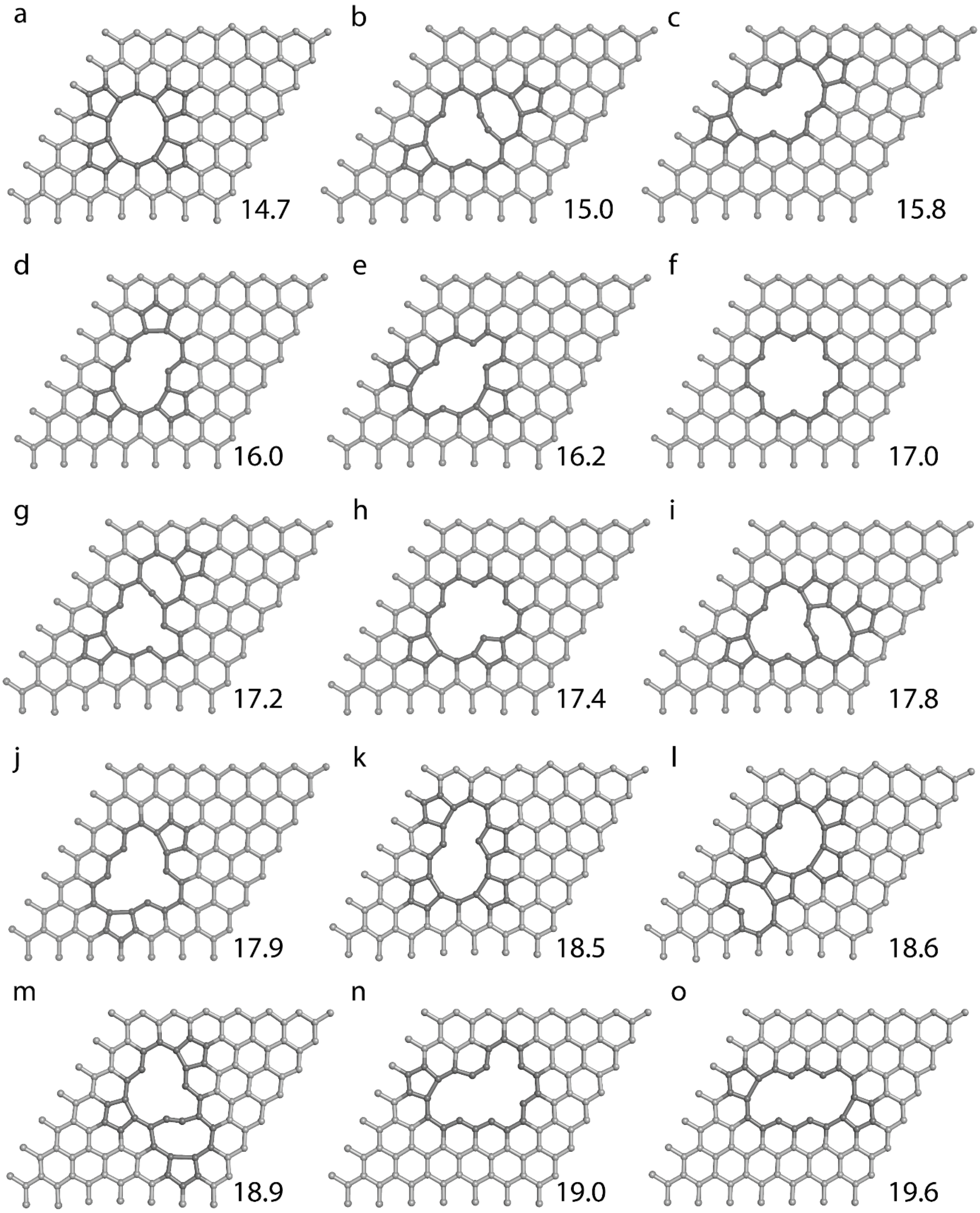

Figure S9: Fifteen most stable optimized defective graphene supercells with sextuple vacancies (six carbon atoms are removed from the lattice of graphene). Numbers are formation energies (E_{form}) in eV. The carbon atoms at the edge of the vacancy are darker for better clarity.

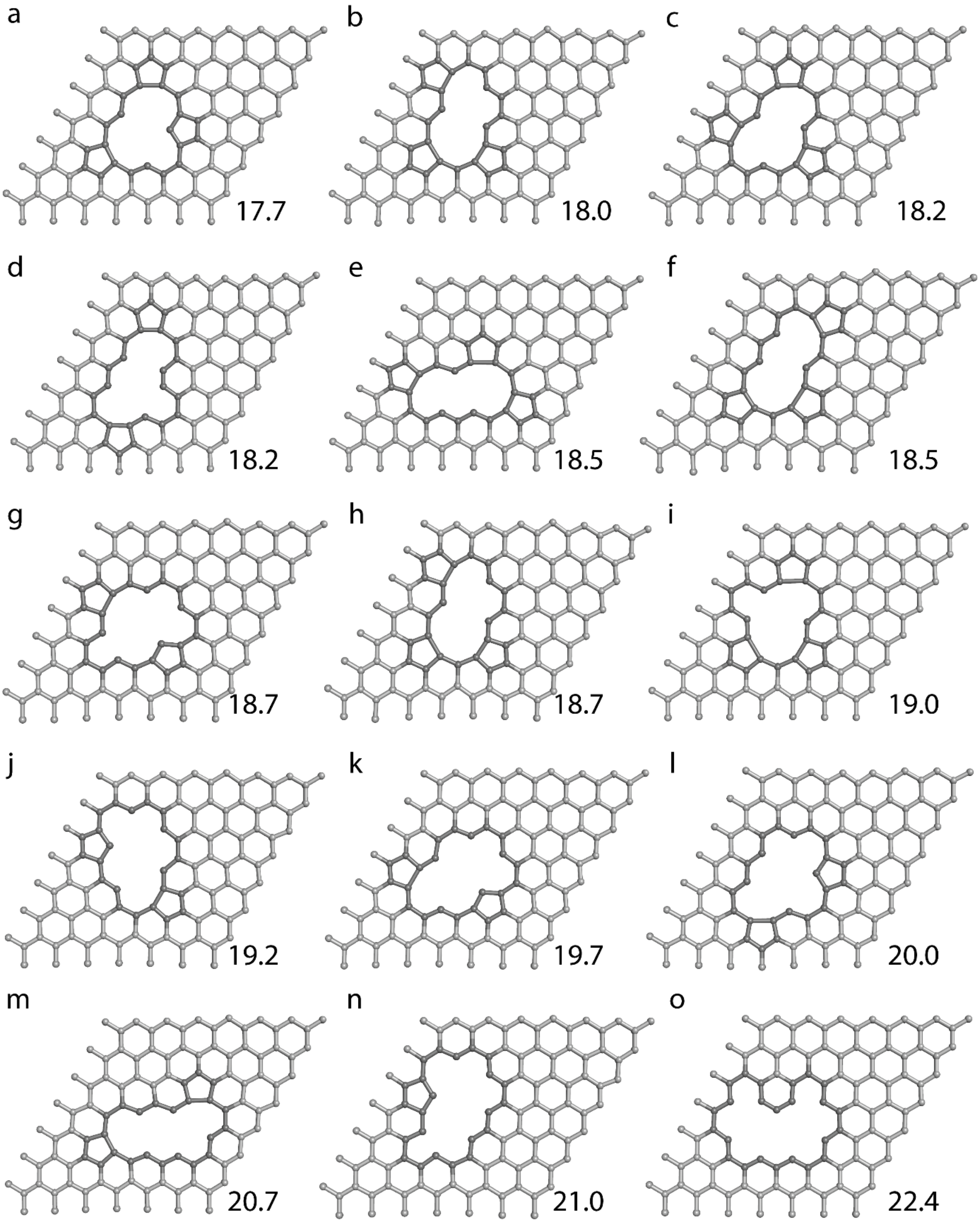

Figure S10: Fifteen most stable optimized defective graphene supercells with septuple vacancies (seven carbon atoms are removed from the lattice of graphene). Numbers are formation energies (E_{form}) in eV. The carbon atoms at the edge of the vacancy are darker for better clarity.

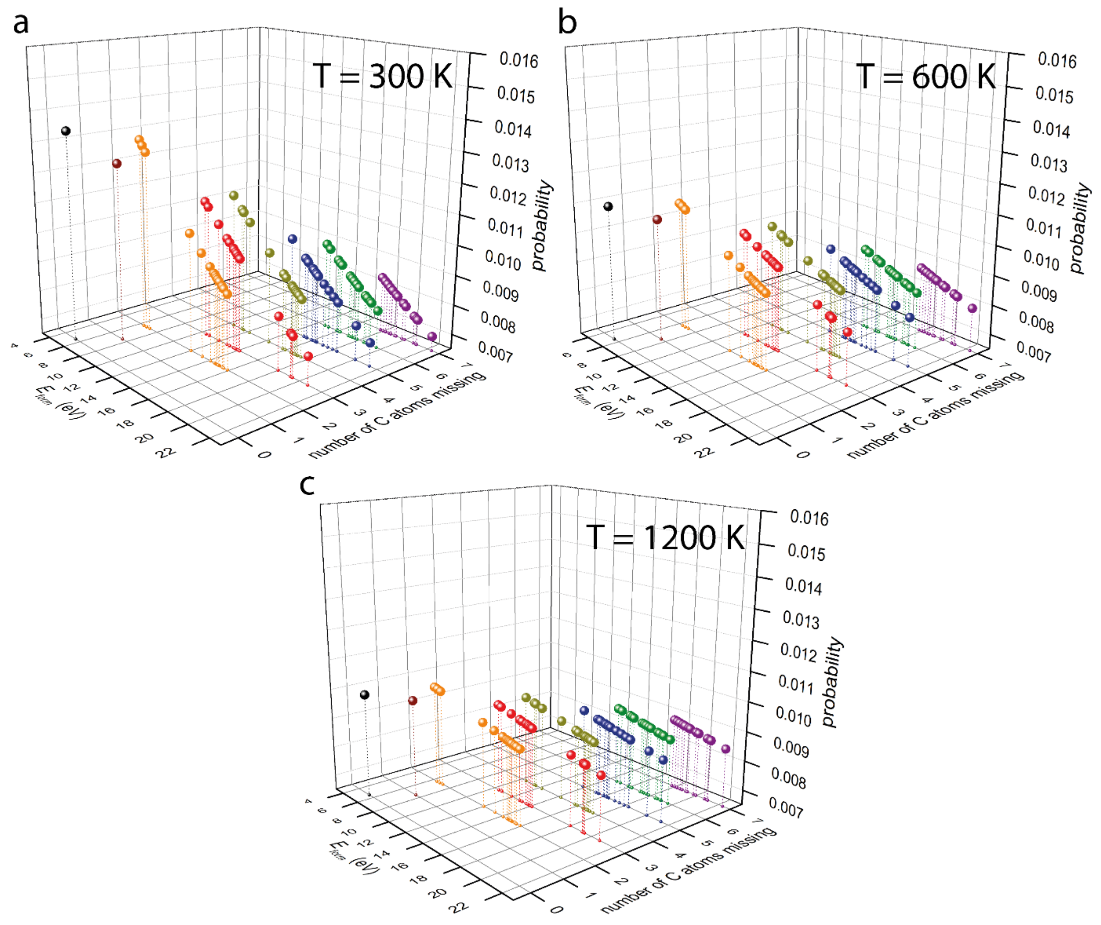

Figure S11: Boltzmann distribution at a) 300 K, b) 600 K, and c) 1200 K and formation energies (E_{form}) of defects in graphene divided into groups according to the size of the vacancy (the number of C atoms missing).

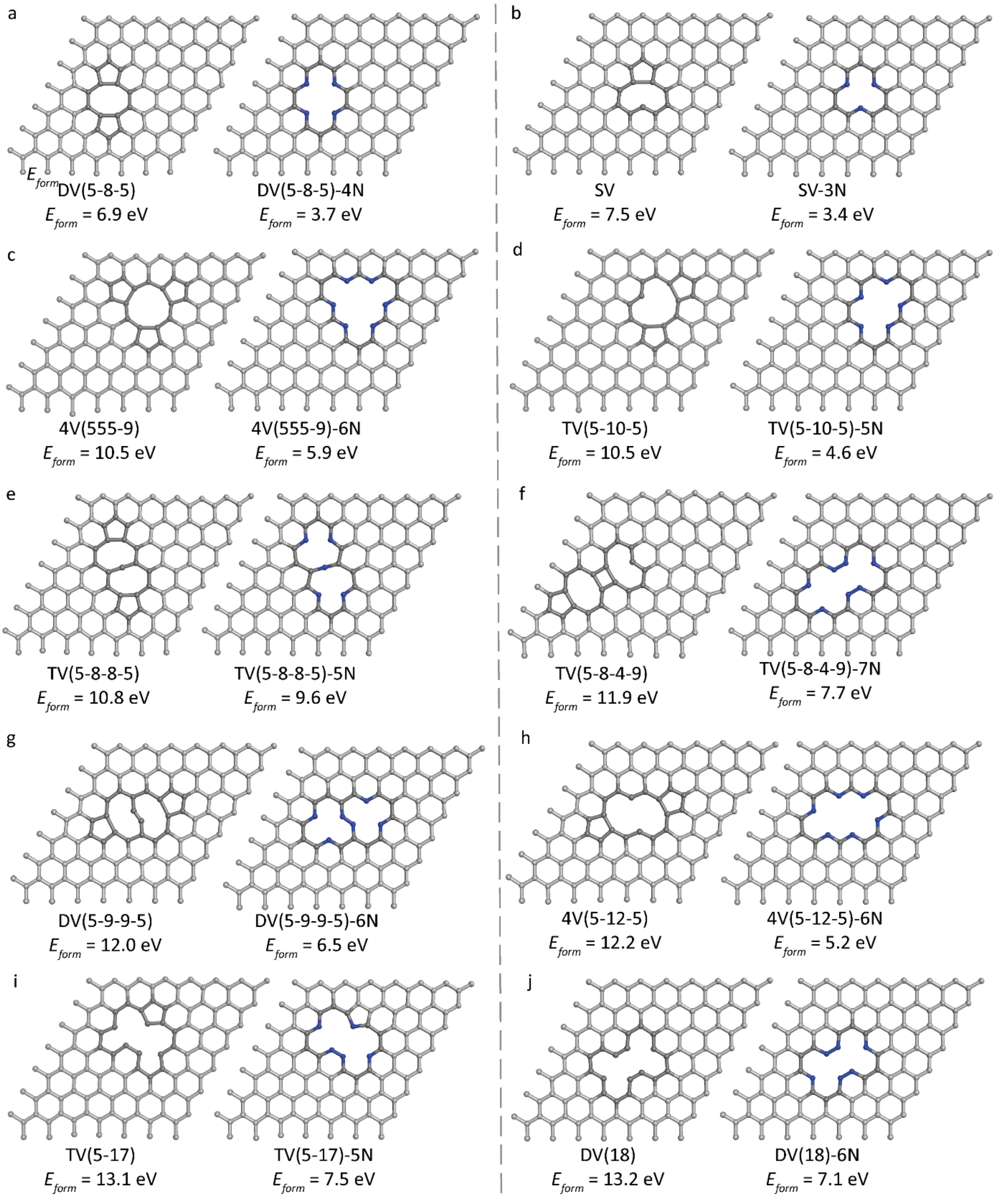

Figure S12: Ten most stable vacancies in undoped graphene and their counterparts with all carbon atoms on the edge of vacancy replaced by nitrogen atoms. Carbon atoms are in grey, nitrogen in blue. The carbon atoms at the edge of the vacancy are darker for better clarity.

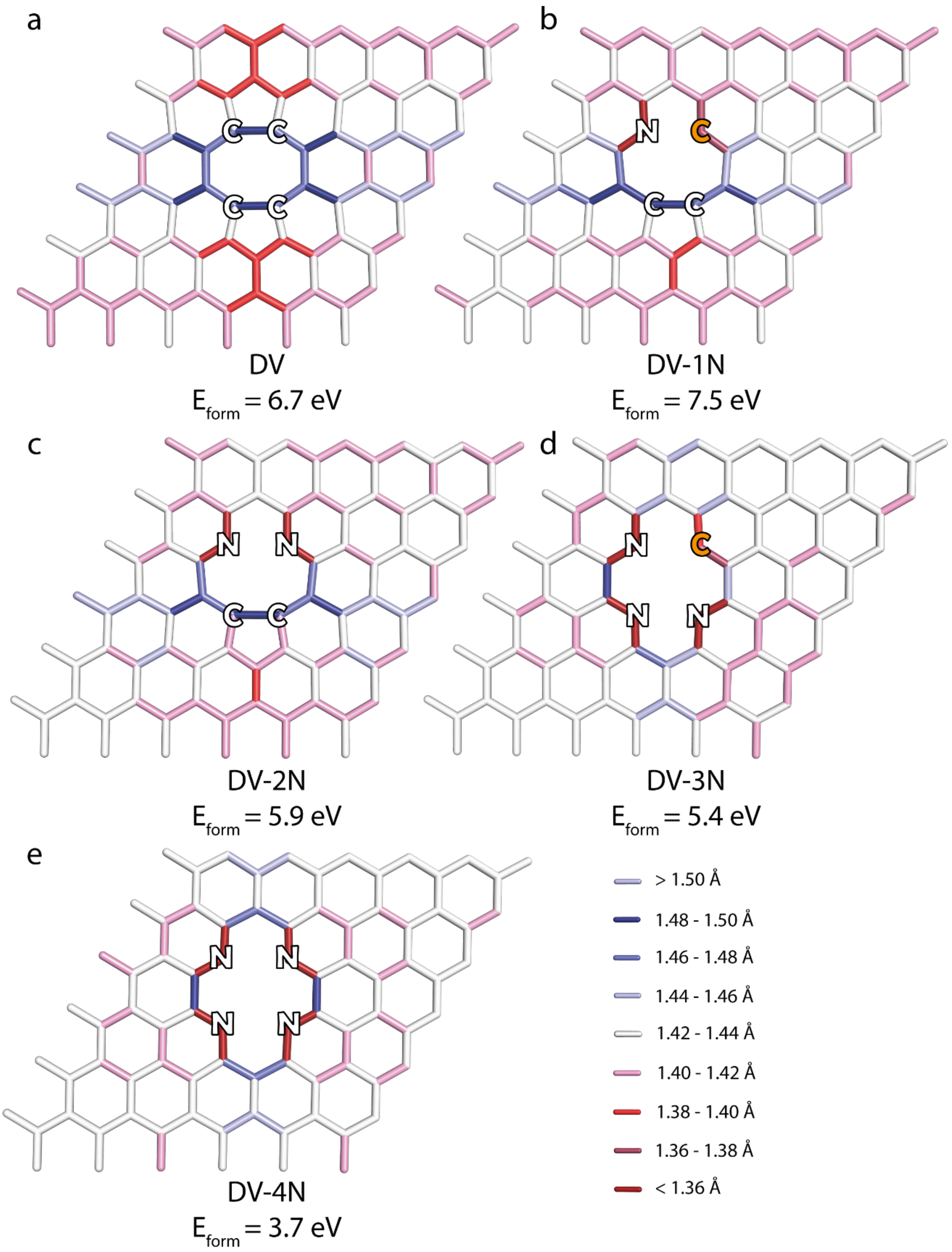

Figure S13: The deformation of the graphene plane with a) DV(5-8-5), b) DV(5-8-5)-1N, c) DV(5-8-5)-2N, d) DV(5-8-5)-3N, and e) DV(5-8-5)-4N defect in the lattice demonstrated by shortening and elongation of the C–C and C–N bonds in comparison to the C–C bond length in pristine graphene (1.43 Å). Carbon atoms with a dangling bond are highlighted in orange.

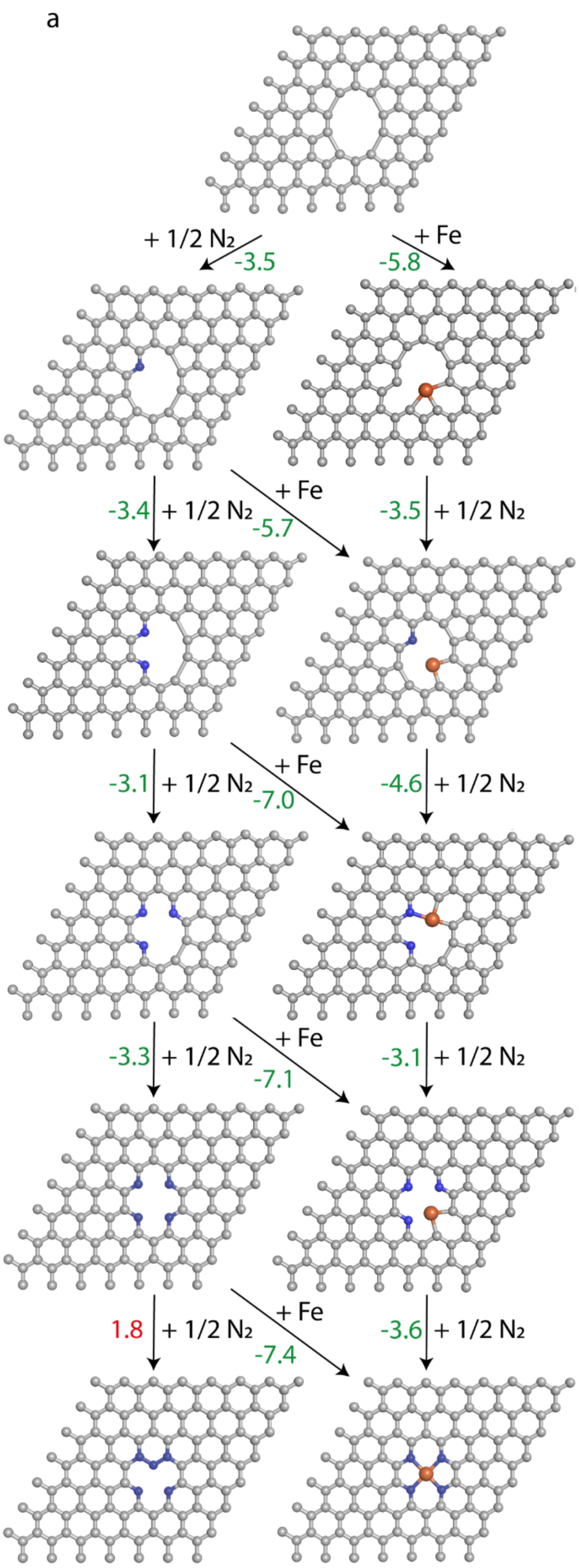

Figure S14: Step by step incorporation of nitrogen atoms and iron atom into A) 6V(5555-10) that leads to the formation of Fe@N_4 and B) V4(5-12-5) that leads to the formation of Fe@C_2N_2. Values are reaction energies (ΔE_R) in eV calculated according to **Eq. S3**. Carbon atoms are grey, nitrogen blue, iron ochre.

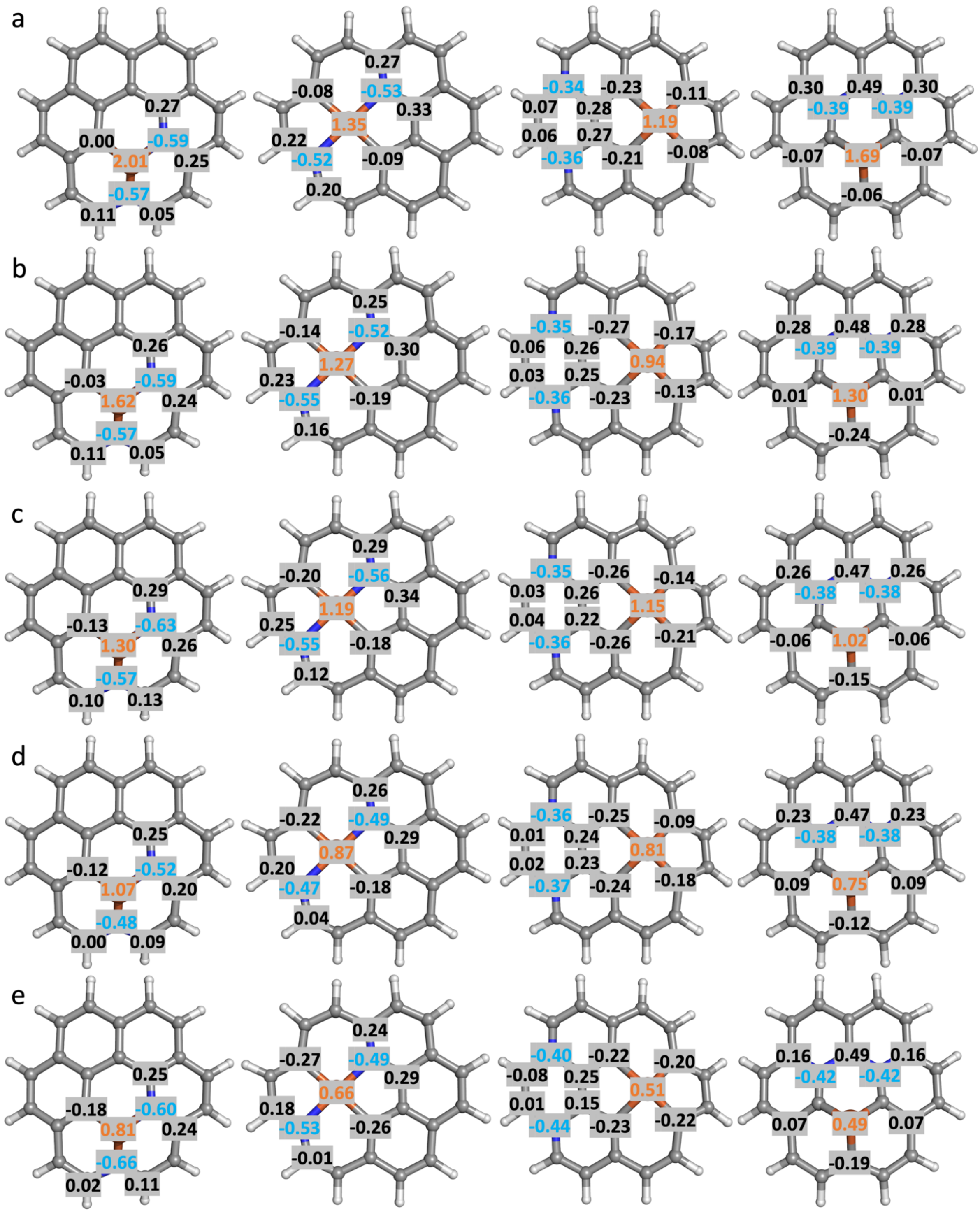

Figure S15: NBO analysis of selected Fe@NG with high Fe stability. In water (a) Fe^{III}, (b) Fe^{II} and in gas phase (c) Fe^{III}, (d) Fe^{II}, (e) Fe^{0}. Numbers depict the NBO charges of individual atoms. Carbon in black, nitrogen in blue, iron in ochre, hydrogen in white.

Table S2: E_{int} (eV) of Fe(III)@N-doped graphene with NN atoms, coronene model. Letters correspond to structures shown in **Figure 4**. Numbers stand for multiplicity of Fe@N-doped graphene. Water implicit solvent.

**structure**	**1**	**2**	**3**	**4**	**5**	**6**	**7**	**8**
	SV							
**a**	-1.34	-	-2.75		-2.72	-	-2.21	-
**b**	-1.65	-	-3.15	-	-2.84	-	-2.85	-
**c**	-	-3.94	-	**-4.97**	-	-4.74	-	-3.95
**d**	-	-2.16	-	-3.56	-	-3.44	-	-2.26
**e**	-3.29	-	-3.79	-	-4.61	-	-3.12	-
**f**	-2.10	-	-3.08	-	-3.30	-	-2.58	-
**g**	-0.83	-	-1.89	-	-2.04	-	-1.24	-
**h**	-	-1.28	-	-2.25	-	-3.32	-	-1.44
**i**	-	-2.68	-	-4.12	-	-3.64	-	-1.42
**j**	-	-3.42	-	-3.18	-	-3.90	-	-3.00
**k**	-	-2.88	-	**-5.12**	-	-4.82	-	-2.87
**l**	-0.74	-	-2.08	-	-2.91	-	-1.25	-
**m**	-0.83	-	-1.73	-	-3.08	-	-0.56	-
**n**	-	-2.97	-	-3.64	-	-1.67	-	-1.70

Table S3: E_{int} (eV) of Fe(II)@N-doped graphene with NN atoms, coronene model. Letters correspond to structures shown in **Figure 4**. Numbers stand for multiplicity of Fe@N-doped graphene. Water implicit solvent.

**structure**	**1**	**2**	**3**	**4**	**5**	**6**	**7**	**8**
	SV							
**a**	-	-1.77	-	-1.52	-	-1.39	-	-0.26
**b**	-	-1.86	-	-2.31	-	-2.30	-	-0.50
**c**	-2.31	-	**-3.88**	-	-3.87	-	-3.33	-
**d**	-0.91	-	-2.35	-	-2.43	-	-1.81	-
**e**	-	-3.08	-	-3.32	-	-3.67	-	-1.95
**f**	-	-1.70	-	-2.79	-	-2.32	-	-0.65
**g**	-	-2.91	-	-2.60	-	-0.88	-	0.98
**h**	-0.61	-	-3.04	-	-3.03	-	-1.20	-
**i**	-1.58	-	-3.40	-	-3.40	-	-1.63	-
**j**	-2.20	-	-4.42	-	-2.93	-	-2.79	-
**k**	-2.48	-	**-4.52**	-	-4.37	-	-2.70	-
**l**	-	-0.99	-	-2.52	-	-1.06	-	0.24
**m**	-	-1.23	-	-2.72	-	-0.84	-	0.45
**n**	-0.36	-	-2.34	-	-2.34	-	-1.30	-

Table S4: E_{int} (eV) of Fe(III)@N-doped graphene with NN atoms, coronene model. Letters correspond to structures shown in **Figure 4**. Numbers stand for multiplicity of Fe@N-doped graphene. Gas phase.

**structure**	**1**	**2**	**3**	**4**	**5**	**6**	**7**	**8**
	SV							
**a**	-30.57	-	-31.21	-	-30.98	-	-30.19	-
**b**	-29.42	-	-30.23	-	-29.80	-	-29.58	-
**c**	-	-30.30	-	-30.36	-	**-30.61**	-	-30.06
**d**	-	-29.63	-	-30.35	-	-30.22	-	-28.51
**e**	-28.67	-	-29.04	-	-29.10	-	-29.83	-
	DV							
**f**	-28.60	-	-30.16	-	-30.13	-	-31.01	-
**g**	-29.86	-	-29.32	-	-30.89	-	-30.16	-
**h**	-	-30.31	-	-31.62	-	-32.59	-	-30.37
**i**	-	-32.74	-	-32.79	-	-32.49	-	-30.10
**j**	-	-33.19	-	-33.33	-	-32.77	-	-32.46
**k**	-	-31.50	-	-32.59	-	**-33.52**	-	-31.23
**l**	-29.61	-	-30.52	-	-31.96	-	-30.84	-
**m**	-30.55	-	-32.52	-	-32.40	-	-30.85	-
**n**	-	-31.59	-	-31.47	-	-31.53	-	-29.08

Table S5: E_{int} (eV) of Fe(II)@N-doped graphene with NN atoms, coronene model. Letters correspond to structures shown in **Figure 4**. Numbers stand for multiplicity of Fe@N-doped graphene. Gas phase.

**structure**	**1**	**2**	**3**	**4**	**5**	**6**	**7**	**8**
	SV							
**a**	-	-14.07	-	-13.64	-	-13.63	-	-10.47
**b**	-	-13.08	-	-13.17	-	-12.92	-	-11.66
**c**	-13.28	-	**-13.60**	-	-13.57	-	-13.55	-
**d**	-12.81	-	-12.14	-	-12.96	-	-12.29	-
**e**	-	-12.31	-	-12.35	-	-12.60	-	-10.34
	DV							
**f**	-	-13.51	-	-14.47	-	-14.50	-	-12.66
**g**	-	-12.64	-	-14.20	-	-13.72	-	-11.98
**h**	-13.55	-	-14.53	-	-16.11	-	-14.29	-
**i**	-14.71	-	-15.35	-	-16.40	-	-14.10	-
**j**	-15.29	-	-16.83	-	-15.98	-	-15.95	-
**k**	-14.87	-	-15.22	-	**-16.88**	-	-14.40	-
**l**	-	-14.43	-	-15.30	-	-14.47	-	-11.29
**m**	-	-14.16	-	-15.77	-	-14.68	-	-12.95
**n**	-14.23	-	-14.82	-	-15.48	-	-12.71	-

Table S6: E_{int} (eV) of Fe(0)@N-doped graphene with NN atoms, coronene model. Letters correspond to structures shown in **Figure 4**. Numbers stand for multiplicity of Fe@N-doped graphene. Gas phase.

**structure**	**1**	**2**	**3**	**4**	**5**	**6**	**7**	**8**
	SV							
**a**	-	**-8.03**	-	-7.81	-	-7.12	-	-4.39
**b**	-	-7.68	-	-7.48	-	-6.76	-	-3.44
**c**	-7.20	-	-7.34	-	-5.75	-	-5.61	-
**d**	-4.02	-	-6.10	-	-6.31	-	-4.68	-
**e**	-	-4.23	-	-5.02	-	-4.96	-	-3.13
	DV							
**f**	-	-8.70	-	-9.64	-	-8.23	-	-5.02
**g**	-	-7.52	-	-8.51	-	-7.33	-	-4.56
**h**	-8.37	-	-9.80	-	-9.37	-	-6.78	-
**i**	-7.96	-	-9.44	-	-9.29	-	-6.88	-
**j**	-9.94	-	-10.30	-	-10.01	-	-7.73	-
**k**	-9.55	-	**-10.98**	-	-10.37	-	-7.66	-
**l**	-	-8.16	-	-8.68	-	-6.68	-	-3.39
**m**	-	-8.20	-	-8.66	-	-6.82	-	-3.92
**n**	-8.03	-	-8.66	-	-7.42	-	-4.87	-

Table S7: E_{int} (eV) of Fe@N-doped graphene with NN atoms, coronene model. Letters correspond to structures in **Figure 4**. Method 1, method 2 and method 3 denote computational methods as described in computational details. Charge 2+ and multiplicity 3 and 4 were considered. Water implicit solvent.

structure	method 1	method 2	method 3
		SV	
**a**	-2.05	-2.07	-1.52
**b**	-1.35	-1.36	-2.31
**c**	**-2.20**	-2.06	**-3.88**
**d**	-2.10	**-2.10**	-2.35
**e**	-0.25	-1.94	-3.32
		DV	
**f**	-2.56	-2.81	-2.79
**g**	-1.17	-1.40	-2.60
**h**	-2.92	-2.89	-3.04
**i**	-3.28	-3.22	-3.40
**j**	-4.04	-3.65	-4.42
**k**	**-4.24**	**-3.85**	**-4.52**
**l**	-2.49	-2.70	-2.52
**m**	-2.70	-2.73	-2.72
**n**	-2.38	-2.43	-2.34

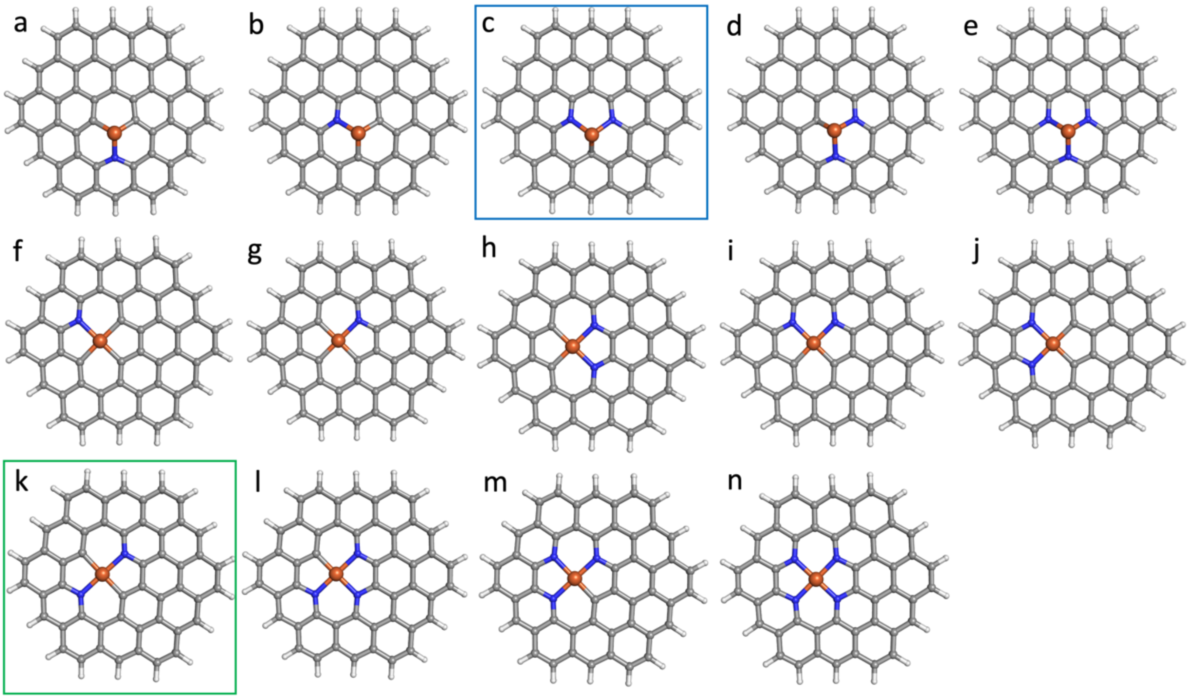

Figure S16: Circumcoronene model of Fe@N-doped graphene with NN atoms. Structures correspond to **Table S8–S12**. Carbon in gray, nitrogen in blue, iron in ochre, hydrogen in white. Blue/green square depicts most stable arrangement of Fe@NSV and Fe@NDV, respectively.

Table S8: E_{int} (eV) of Fe(III)@N-doped graphene with NN atoms, circumcoronene model. Letters correspond to structures in **Figure S16**. Numbers stand for multiplicity of Fe@N-doped graphene with odd/even number of nitrogen atoms. Water implicit solvent.

structure	1	2	3	4	5	6	7	8
	SV							
**a**	-1.65	-	-2.46	-	-1.61	-	-1.02	-
**b**	-1.25	-	-2.14	-	-1.79	-	-1.22	-
**c**	-	-2.47	-	-2.70	-	**-2.87**	-	-2.43
**d**	-	-1.48	-	-1.87	-	-1.93	-	-1.48
**e**	-1.73	-	-1.87	-	-1.84	-	-1.52	-
	DV							
**f**	-1.23	-	-2.59	-	-3.96	-	-3.58	-
**g**	-0.69	-	-2.98	-	-3.69	-	-3.53	-
**h**	-	-3.65	-	-4.21	-	-4.19	-	-3.19
**i**	-	-3.35	-	-3.94	-	-3.94	-	-2.46
**j**	-	-4.69	-	-5.45	-	-5.02	-	-3.67
**k**	-	-5.28	-	-5.08	-	**-5.48**	-	-4.00
**l**	-1.52	-	-2.36	-	-2.36	-	-2.30	-
**m**	-2.17	-	-4.23	-	-4.23	-	-2.46	-
**n**	-	-3.24	-	-3.98	-	-3.26	-	-2.06

Table S9: E_{int} (eV) of Fe(II)@N-doped graphene with NN atoms, circumcoronene model. Letters correspond to structures in **Figure S16**. Numbers stand for multiplicity of Fe@N-doped graphene with odd/even number of nitrogen atoms. Water implicit solvent.

**structure**	**1**	**2**	**3**	**4**	**5**	**6**	**7**	**8**
	SV							
**a**	-	-0.44	-	-0.25	-	0.31	-	1.69
**b**	-	-0.52	-	-0.41	-	0.17	-	2.15
**c**	-0.86	-	**-1.47**	-	-1.38	-	-1.23	-
**d**	0.03	-	0.21	-	-0.39	-	-0.05	-
**e**	-	-0.48	-	-0.18	-	-0.48	-	1.63
**f**	-	-1.33	-	-3.03	-	-2.39	-	-0.80
**g**	-	-1.30	-	-2.62	-	-2.43	-	-0.85
**h**	-0.95	-	-1.11	-	-3.13	-	-2.02	-
**i**	-0.55	-	-1.44	-	-3.14	-	-1.10	-
**j**	-2.52	-	-4.15	-	-3.92	-	-2.50	-
**k**	-2.26	-	**-4.62**	-	-4.42	-	-2.68	-
**l**	-	-1.04	-	-2.81	-	-0.45	-	1.24
**m**	-	-1.07	-	-2.85	-	-1.03	-	1.38
**n**	-0.50	-	-2.36	-	-2.01	-	-0.64	-

Table S10: E_{int} (eV) of Fe(III)@N-doped graphene with NN atoms, circumcoronene model. Letters correspond to structures in **Figure S16**. Numbers stand for multiplicity of Fe@N-doped graphene with odd/even number of nitrogen atoms. Gas phase.

**structure**	**1**	**2**	**3**	**4**	**5**	**6**	**7**	**8**
	SV							
**a**	-33.96	-	-34.54	-	-33.70	-	-33.92	-
**b**	-33.51	-	-34.42	-	-33.92	-	-33.21	-
**c**	-	**-34.86**	**-**	-33.85	-	-34.76	-	-32.86
**d**	-	-33.68	-	-33.82	-	-33.82	-	-33.28
**e**	-33.82	-	-33.82	-	-33.55	-	-33.77	-
	DV							
**f**	-33.66	-	-34.37	-	-36.53	-	-36.29	-
**g**	-33.81	-	-33.79	-	-35.39	-	-35.90	-
**h**	-	-35.95	-	-36.13	-	-37.46	-	-35.91
**i**	-	-35.22	-	-37.04	-	-37.08	-	-35.48
**j**	-	-37.98	-	-38.44		-38.06	-	-36.04
**k**	-	-37.85	-	-38.46	-	**-38.59**	-	-36.82
**l**	-33.98	-	-35.26	-	-36.58	-	-35.51	-
**m**	-35.09	-	-36.25	-	-36.77	-	-35.41	-
**n**	-	-36.56	-	-36.98	-	-35.62	-	-33.31

Table S11: E_{int} (eV) of Fe(II)@N-doped graphene with NN atoms, circumcoronene model. Letters correspond to structures in **Figure S16**. Numbers stand for multiplicity of Fe@N-doped graphene with odd/even number of nitrogen atoms. Gas phase.

structure	1	2	3	4	5	6	7	8
	SV							
**a**	-	-14.60	-	-14.49	-	-13.90	-	-11.61
**b**	-	-14.27	-	-14.49	-	-13.96	-	-11.89
**c**	-14.68	-	**-15.37**	-	-15.34	-	-13.44	-
**d**	-13.98	-	-13.89	-	-14.36	-	-13.30	-
**e**	-	-14.18	-	-13.94	-	-13.75	-	-12.53
	DV							
**f**	-	-16.25	-	-17.26	-	-16.80	-	-14.94
**g**	-	-17.11	-	-16.14	-	-16.55	-	-14.94
**h**	-15.27	-	-16.85	-	-17.47	-	-16.54	-
**i**	-15.24	-	-16.38	-	-17.81	-	-16.28	-
**j**	-16.42	-	-18.86	-	-18.12	-	-17.36	-
**k**	-16.39	-	-18.21	-	**-19.25**	-	-17.82	-
**l**	-	-15.80	-	-17.13	-	-16.22	-	-13.60
**m**	-	-16.25	-	-17.22	-	-16.06	-	-13.41
**n**	-15.89	-	-17.52	-	-16.18	-	-14.00	-

Table S12: E_{int} (eV) of Fe(0)@N-doped graphene with NN atoms, circumcoronene model. Letters correspond to structures shown in **Figure S16**. Numbers stand for multiplicity of Fe@N-doped graphene. Gas phase.

structure	1	2	3	4	5	6	7	8
	SV							
**a**	-	-6.36	-	-5.64	-	-4.59	-	-2.19
**b**	-	-6.62	-	-4.77	-	-4.45	-	-2.29
**c**	**-6.64**	-	-6.49	-	-6.50	-	-5.05	-
**d**	-5.13	-	-5.01	-	-5.07	-	-3.74	-
**e**	-	-4.43	-	-4.05	-	-4.46	-	-2.81
	DV							
**f**	-	-8.37	-	-9.46	-	-8.33	-	-6.04
**g**	-	-8.31	-	-7.54	-	-8.16	-	-5.95
**h**	-7.89	-	-9.47	-	-9.59	-	-7.11	-
**i**	-7.40	-	-9.01	-	-8.65	-	-6.79	-
**j**	-8.13	-	-10.44	-	-9.63	-	-7.91	-
**k**	-9.16	-	**-10.78**	-	-10.41	-	-8.29	-
**l**	-	-6.75	-	-8.18	-	-6.71	-	-2.59
**m**	-	-7.93	-	-7.07	-	-6.39	-	-3.84
**n**	-6.50	-	-8.01	-	-6.86	-	-4.90	-

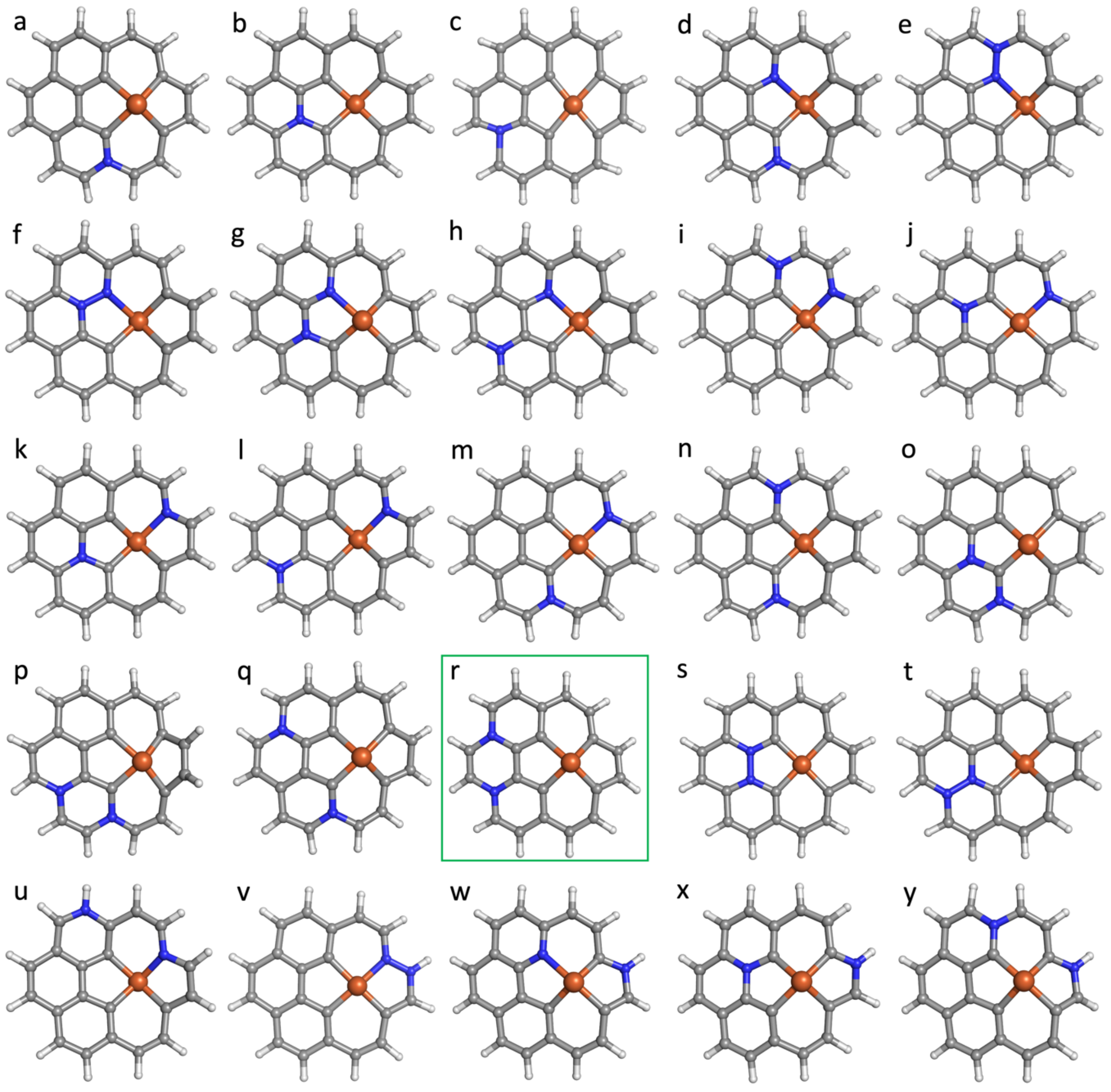

Figure S17: Coronene model of Fe@N-doped graphene with NNN and DN atoms. Structures correspond to **Table S13 and Table S14**. Carbon in gray, nitrogen in blue, iron in ochre, hydrogen in white. Green square depicts most stable arrangement of Fe@NDV.

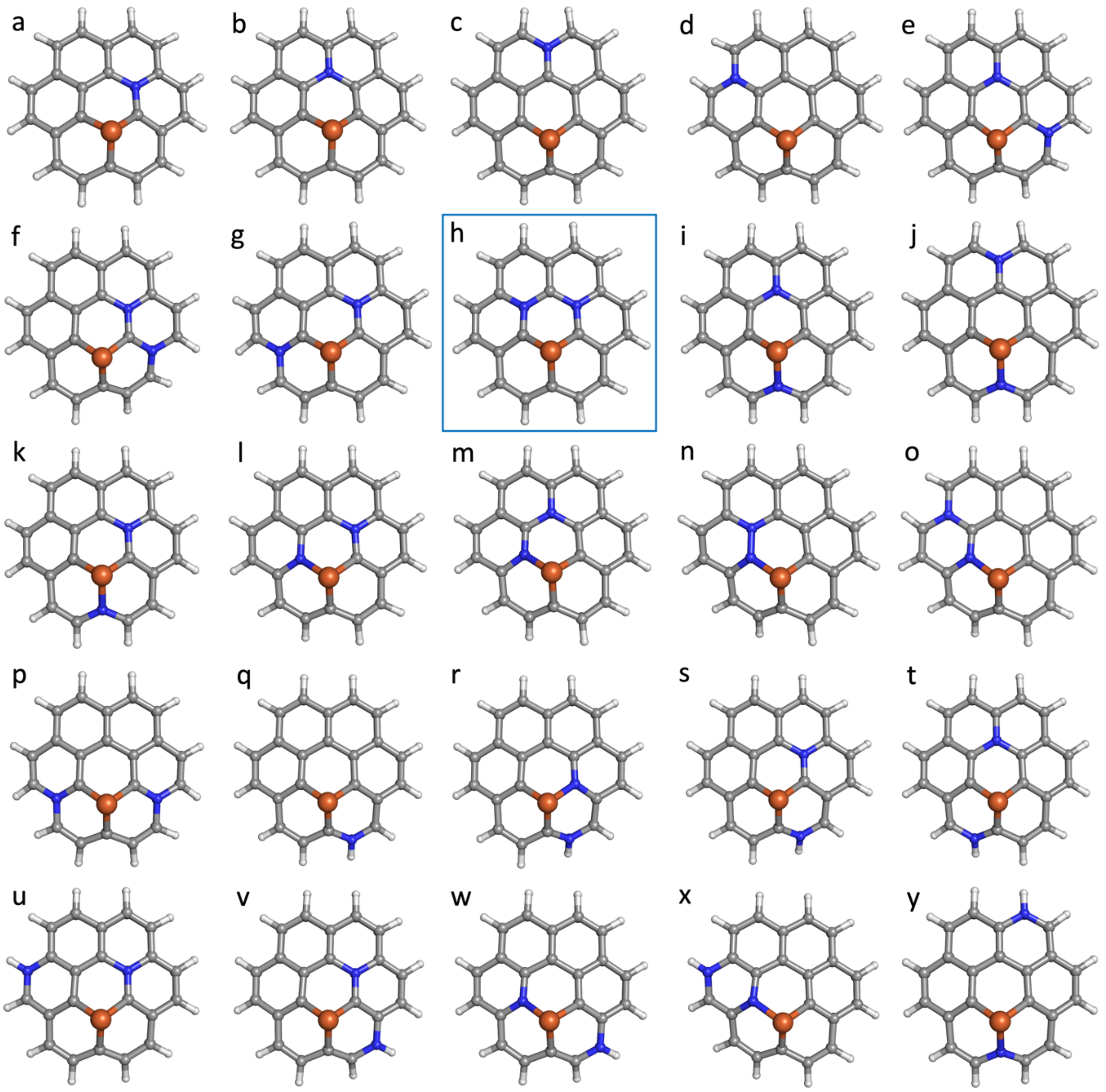

Figure S18: Coronene model of Fe@N-doped graphene with NNN and DN atoms. Structures correspond to **Table S13** and **Table S14**. Carbon in gray, nitrogen in blue, iron in ochre, hydrogen in white. Blue square depicts most stable arrangement of Fe@NSV.

Table S13: E_{int} (eV) of Fe@N-doped graphene with NNN and DN atoms, coronene model. Letters correspond to structures in **Figure S17** (Fe@NDV) and **Figure S18** (Fe@NSV). Water implicit solvent.

**structure**	**DV**		**SV**	
	**Fe^{3+}**	**Fe^{2+}**	**Fe^{3+}**	**Fe^{2+}**
**a**	-5.86	-4.77	-3.31	-2.69
**b**	-6.54	-5.43	-3.54	-2.51
**c**	-6.76	-5.54	-4.24	-3.16
**d**	-5.32	-2.79	-3.42	-2.50
**e**	-5.28	-3.73	-4.00	-3.91
**f**	-5.57	-2.92	-3.59	-2.93
**g**	-5.44	-2.95	-3.50	-2.41
**h**	-5.63	-4.13	**-5.14**	**-4.02**
**i**	-4.72	-3.13	-3.37	-2.54
**j**	-4.57	-2.51	-3.53	-2.74
**k**	-5.49	-4.30	-3.41	-2.24
**l**	-5.60	-4.20	-2.23	-1.86
**m**	-5.00	-3.38	-3.57	-2.15
**n**	-6.32	-4.74	-2.28	-1.71
**o**	-6.38	-4.77	-2.66	-2.27
**p**	-6.66	-4.98	-3.81	-3.02
**q**	-6.67	-4.97	-2.31	-1.79
**r**	**-6.96**	**-5.59**	-2.67	-1.76
**s**	-6.91	-5.13	-4.10	-3.31
**t**	-6.55	-4.89	-3.26	-2.71
**u**	-5.43	-3.83	-4.35	-3.75
**v**	-5.27	-2.83	-3.21	-2.95
**w**	-5.30	-2.54	-2.08	-2.05
**x**	-6.15	-4.70	-0.67	-0.02
**y**	-6.07	-2.73	-3.63	-1.18

Table S14: E_{int} (eV) of Fe@N-doped graphene with NNN and DN atoms, coronene model. Letters correspond to structures in **Figure S17** (Fe@NDV) and **Figure S18** (Fe@NSV). Gas phase.

**structure**	**DV**			**SV**		
	**Fe^{3+}**	**Fe^{2+}**	**Fe^{0}**	**Fe^{3+}**	**Fe^{2+}**	**Fe^{0}**
**a**	-33.63	-17.15	-10.01	-31.78	-14.93	-7.64
**b**	-33.46	-16.97	-10.10	-32.42	-15.49	-7.96
**c**	-32.04	-15.22	-8.82	-32.62	-15.81	-7.93
**d**	-33.42	-16.65	-9.05	-31.97	-15.15	-7.78
**e**	-33.67	-16.90	-10.03	-34.22	-16.66	-8.37
**f**	-33.78	-16.68	-9.28	-33.37	-16.16	-8.07
**g**	-33.50	-17.21	-9.77	-34.37	-17.15	-8.34
**h**	-34.26	-17.43	-9.80	**-35.27**	**-18.38**	**-9.82**
**i**	-33.36	-16.90	-10.37	-31.44	-14.75	-7.01
**j**	-33.66	-17.11	-10.50	-31.95	-15.15	-7.39
**k**	-33.50	-16.83	-10.28	-31.37	-14.63	-7.34
**l**	-33.89	-17.41	-10.29	-32.01	-15.06	-7.27
**m**	-33.74	-16.88	-9.68	-31.40	-14.47	-6.65
**n**	-34.24	-17.76	-10.26	-31.21	-14.30	-6.71
**o**	-34.40	-17.77	-10.50	-31.10	-14.30	-6.64
**p**	-33.76	-17.14	-9.77	-33.77	-16.74	-8.17
**q**	-35.17	-18.44	**-10.51**	-32.23	-15.30	-8.49
**r**	**-35.41**	**-18.47**	**-10.51**	-31.40	-14.47	-7.38
**s**	-34.82	-18.27	-10.41	-33.76	-16.40	-8.10
**t**	-34.52	-17.70	-10.32	-33.95	-16.46	-8.00
**u**	-34.10	-17.23	-10.39	-34.16	-16.54	-7.39
**v**	-33.60	-16.79	-9.71	-32.87	-15.96	-8.01
**w**	-33.05	-16.48	-9.85	-30.75	-13.78	-6.71
**x**	-33.96	-17.36	-10.50	-31.90	-14.86	-7.47
**y**	-33.91	-17.23	-10.36	-32.46	-15.26	-7.27

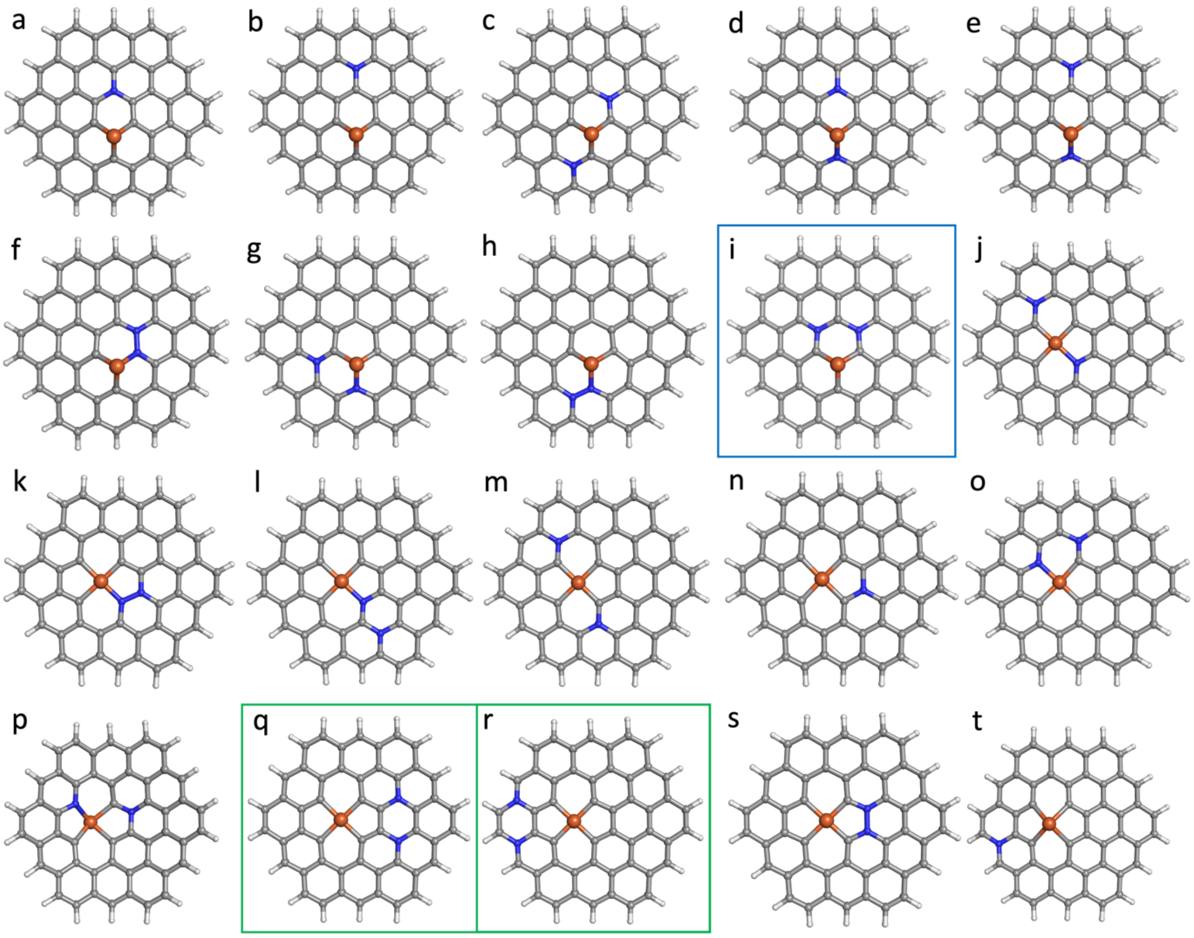

Figure S19: Circumcoronene model of Fe@N-doped graphene with NNN and DN atoms. Structures correspond to **Table S15** and **Table S16**. Carbon in gray, nitrogen in blue, iron in ochre, hydrogen in white. Blue/green square depicts most stable arrangement of Fe@NSV and Fe@NDV, respectively.

Table S15: E_{int} (eV) of Fe@N-doped graphene with NNN and DN atoms, circumcoronene model. Letters correspond to structures in **Figure S19**. Water implicit solvent.

**structure**	**SV**	
	**Fe^{3+}**	**Fe^{2+}**
**a**	-1.89	-0.66
**b**	-3.21	-1.64
**c**	-3.74	-3.00
**d**	-1.80	-0.45
**e**	-2.70	-1.37
**f**	-2.10	-0.96
**g**	-2.65	-1.87
**h**	-3.08	-1.58
**i**	**-4.66**	**-4.43**
	**DV**	
**j**	-5.69	-4.48
**k**	-5.33	-3.91
**l**	-5.42	-4.01
**m**	-6.32	-5.12
**n**	-5.70	-4.34
**o**	-6.16	-4.29
**p**	-4.95	-3.00
**q**	-7.07	**-5.62**
**r**	**-7.27**	-5.20
**s**	-5.95	-3.36
**t**	-4.45	-4.23

Table S16: E_{int} (eV) of Fe@N-doped graphene with NNN and DN atoms, circumcoronene model. Letters correspond to structures in **Figure S19**. Gas phase.

**structure**	**SV**		
	**Fe^{3+}**	**Fe^{2+}**	**Fe^{0}**
**a**	-34.87	-15.06	-7.61
**b**	-36.37	-16.88	-7.98
**c**	-37.44	-17.67	-7.82
**d**	-34.85	-15.09	-6.27
**e**	-35.90	-16.26	-6.51
**f**	-34.56	-15.15	-6.50
**g**	-35.65	-15.86	-6.58
**h**	-35.50	-15.42	-5.84
**i**	**-38.56**	**-19.22**	**-9.58**
	**DV**		
**j**	-38.03	-19.10	-10.57
**k**	-37.76	-18.03	-11.02
**l**	-38.06	-18.77	-10.18
**m**	-39.25	-20.03	-10.51
**n**	-37.10	-18.11	-10.50
**o**	-37.83	-18.64	-9.61
**p**	-38.22	-17.36	-9.92
**q**	**-40.51**	-20.76	**-12.08**
**r**	-40.07	**-21.00**	-10.95
**s**	-37.84	-18.62	-10.84
**t**	-37.43	-18.37	-10.21

Table S17: Electrochemical CO_2RR catalyzed by Fe(III)@NG in water solvent, multiplicity of 1–8. Energy barriers in kcal/mol. Note that 1 kcal/mol ≈ 4.184 kJ/mol ≈ 0.043 eV.

structure	reaction step	1, 2	3, 4	5, 6	7, 8
Fe@ND(S)V (**Figure S17r**)	$*CO_2$	-33.25	1.58	1.21	53.82
	*COOH(TS)	55.53	33.83	33.96	-43.08
	*COOH	49.67	31.45	32.06	-53.40
	*CO	-44.18	-30.57	-37.47	-4.81
	* + CO	21.80	-5.26	-1.76	-1.57
$Fe@N_4$ (**Figure 4n**)	$*CO_2$	-0.47	3.64	-26.54	1.59
	*COOH(TS)	40.96	78.58	30.67	38.91
	*COOH	29.15	43.31	29.32	18.08
	*CO	-39.24	-48.17	-47.09	-14.13
	* + CO	4.60	-4.74	38.36	-11.50
$Fe@C_2N_2$ (**Figure 4k**)	$*CO_2$	-23.50	-1.04	-0.08	1.63
	*COOH(TS)	43.78	32.92	27.41	34.11
	*COOH	43.02	30.12	-36.61	-23.09
	*CO	-22.82	-31.09	34.80	24.55
	* + CO	-2.66	-3.95	-3.93	-9.05

Table S18: Electrochemical CO_2RR catalyzed by Fe(II)@NG in water solvent, multiplicity of 1–8. Energy barriers in kcal/mol.

structure	reaction step	1, 2	3, 4	5, 6	7, 8
Fe@ND(S)V (**Figure S17r**)	$*CO_2$	-6.05	-2.08	0.03	0.13
	*COOH(TS)	6.51	5.39	44.19	71.07
	*COOH	2.68	-27.82	40.26	67.17
	*CO	-16.46	23.62	-39.46	-71.64
	* + CO	13.88	0.32	-6.79	-1.62
$Fe@N_4$ (**Figure 4n**)	$*CO_2$	0.10	-0.04	0.10	2.96
	*COOH(TS)	15.99	39.42	56.91	57.48
	*COOH	11.17	33.43	53.79	41.77
	*CO	-24.35	-35.53	-65.72	-44.99
	* + CO	7.12	-3.81	5.87	-5.71
$Fe@C_2N_2$ (**Figure 4k**)	$*CO_2$	-1.47	-0.67	-1.26	-27.99
	*COOH(TS)	10.07	17.02	45.21	47.85
	*COOH	9.60	-21.36	43.52	39.58
	*CO	-18.80	17.90	-46.02	-39.69
	* + CO	4.71	-4.18	-3.89	22.14

Table S19: CO_2RR catalyzed by Fe(III)@NG in gas phase, multiplicity of 1–8. Energy barriers in kcal/mol.

structure	reaction step	1, 2	3, 4	5, 6	7, 8
Fe@ND(S)V (**Figure S17r**)	*CO_2	-52.75	-16.10	-20.81	-18.14
	*COOH(TS)	61.88	43.05	34.92	14.45
	*COOH	49.98	32.03	30.96	8.83
	*CO	-23.47	-15.49	-12.23	10.25
	* + CO	41.10	14.42	16.94	13.91
Fe@N_4 (**Figure 4n**)	*CO_2	-40.89	-18.58	-16.18	-26.12
	*COOH(TS)	49.75	40.53	40.06	39.58
	*COOH	41.61	32.50	31.22	24.35
	*CO	-18.06	-12.97	-11.19	-3.10
	* + CO	32.20	13.91	11.01	19.73
Fe@C_2N_2 (**Figure 4k**)	*CO_2	-16.32	-14.96	-13.53	-32.80
	*COOH(TS)	43.61	37.84	42.34	33.85
	*COOH	36.53	36.25	33.97	32.00
	*CO	-0.48	-10.14	-17.99	-12.03
	* + CO	-4.87	3.71	3.50	27.68

Table S20: CO_2RR catalyzed by Fe(II)@NG in gas phase, multiplicity of 1–8. Energy barriers in kcal/mol.

structure	reaction step	1, 2	3, 4	5, 6	7, 8
Fe@ND(S)V (**Figure S17r**)	*CO_2	-5.54	-4.24	-7.41	-11.67
	*COOH(TS)	28.61	42.22	79.22	74.30
	*COOH	8.28	34.73	46.01	41.43
	*CO	2.33	-22.41	-32.44	-24.18
	* + CO	9.79	6.77	8.69	9.27
Fe@N_4 (**Figure 4n**)	*CO_2	-24.79	-5.47	-8.75	-10.22
	*COOH(TS)	34.14	51.74	51.89	44.20
	*COOH	23.55	41.32	33.37	41.73
	*CO	-14.06	-25.44	-14.57	-25.72
	* + CO	30.16	4.45	4.80	9.07
Fe@C_2N_2 (**Figure 4k**)	*CO_2	-11.33	-11.15	-7.45	-5.97
	*COOH(TS)	21.93	46.15	53.02	49.04
	*COOH	12.90	39.74	46.68	44.41
	*CO	-0.95	-16.87	-32.56	-29.34
	* + CO	14.23	3.14	8.20	5.75

Table S21: CO_2RR catalyzed by Fe(0)@NG in gas phase, multiplicity of 1–8. Energy barriers in kcal/mol.

structure	reaction step	1, 2	3, 4	5, 6	7, 8
Fe@ND(S)V (**Figure S17r**)	*CO_2	-6.80	-1.09	-0.32	0.25
	*COOH(TS)	39.87	51.13	48.48	50.15
	*COOH	30.59	33.12	33.93	47.12
	*CO	-27.16	-20.67	-22.11	-30.16
	* + CO	18.22	3.50	3.35	-2.35
Fe@N_4 (**Figure 4n**)	*CO_2	-21.33	0.31	-18.32	0.32
	*COOH(TS)	17.12	52.65	27.93	48.73
	*COOH	12.62	40.35	23.74	31.79
	*CO	-10.02	-22.71	11.22	-18.27
	* + CO	33.59	-3.10	-1.79	1.01
Fe@C_2N_2 (**Figure 4k**)	*CO_2	-21.23	-2.06	-1.11	-23.98
	*COOH(TS)	16.92	41.65	61.44	77.37
	*COOH	12.40	21.96	35.17	35.02
	*CO	-13.54	-13.27	-19.12	-20.55
	* + CO	37.22	8.22	-0.07	24.36
